# Elucidating dynamic anaerobe metabolism with HRMAS ^13^C NMR and genome-scale modeling

**DOI:** 10.1038/s41589-023-01275-9

**Published:** 2023-03-09

**Authors:** Aidan Pavao, Brintha Girinathan, Johann Peltier, Pamela Altamirano Silva, Bruno Dupuy, Isabella H. Muti, Craig Malloy, Leo L. Cheng, Lynn Bry

**Affiliations:** 1 Massachusetts Host-Microbiome Center, Brigham and Women’s Hospital, Harvard Medical School, Boston, MA USA; 2 Laboratoire Pathogenèse des Bactéries Anaérobies, F-75015, Institut Pasteur, Université Paris-Cité, UMR-CNRS 6047, Paris, France; 3 Institute for Integrative Biology of the Cell (I2BC), 91198, University of Paris-Saclay, CEA, CNRS, Gif-sur-Yvette, France; 4 Centre for Investigations in Tropical Diseases, Faculty of Microbiology, University of Costa Rica, San José, Costa Rica; 5 Departments of Radiology and Pathology, Massachusetts General Hospital, Harvard Medical School, Boston, MA USA; 6 Department of Radiology, The University of Texas Southwestern Medical Center, Dallas, TX USA; 7 Clinical Microbiology Laboratory, Department of Pathology, Brigham and Women’s Hospital, Harvard Medical School, Boston, MA USA; 8 Present Address: Ginkgo Bioworks, The Innovation and Design Building, Boston, MA USA

**Keywords:** Bacteria, Biochemical reaction networks, Networks and systems biology, Metabolic pathways

## Abstract

Anaerobic microbial metabolism drives critical functions within global ecosystems, host–microbiota interactions, and industrial applications, yet remains ill-defined. Here we advance a versatile approach to elaborate cellular metabolism in obligate anaerobes using the pathogen *Clostridioides difficile*, an amino acid and carbohydrate-fermenting *Clostridia*. High-resolution magic angle spinning nuclear magnetic resonance (NMR) spectroscopy of *C. difficile*, grown with fermentable ^13^C substrates, informed dynamic flux balance analysis (dFBA) of the pathogen’s genome-scale metabolism. Analyses identified dynamic recruitment of oxidative and supporting reductive pathways, with integration of high-flux amino acid and glycolytic metabolism at alanine’s biosynthesis to support efficient energy generation, nitrogen handling and biomass generation. Model predictions informed an approach leveraging the sensitivity of ^13^C NMR spectroscopy to simultaneously track cellular carbon and nitrogen flow from [U-^13^C]glucose and [^15^N]leucine, confirming the formation of [^13^C,^15^N]alanine. Findings identify metabolic strategies used by *C. difficile* to support its rapid colonization and expansion in gut ecosystems.

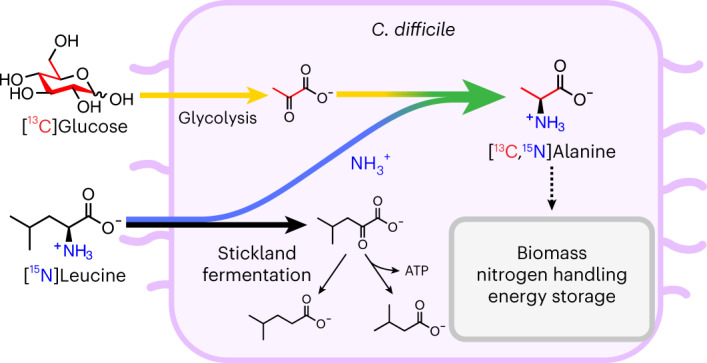

## Abstract (web summary)

Live-cell HRMAS NMR spectroscopy and genome-scale metabolic modeling enable high-resolution analysis of dynamic, anaerobic metabolism in *Clostridioides difficile*, identifying the confluence of carbohydrate and amino acid fermentations for alanine synthesis.

## Main

Obligate anaerobes comprise the majority of species in the mammalian gut microbiota and include pathogens such as *Clostridioides* *difficile*. Anaerobic bacteria also modulate nutrient flow across global ecosystems^1^ and perform industrial fermentations of economic importance^2^. However, the metabolic pathways and nutrient requirements of anaerobes often differ substantively from those of model, aerotolerant species such as *Escherichia coli* or *Bacillus subtilis*, leaving many aspects of their metabolism poorly defined. This poor characterization limits efforts to harness anaerobe metabolism in applications of clinical, industrial or environmental importance.

*C. difficile*, the leading cause of hospital-acquired infections, colonizes gut environments through its fermentation of diverse carbon sources^3^, including carbohydrates and amino acids^4–6^. The pathogen releases toxins to obtain nutrients from damaged mucosa as its growth exceeds the carrying capacity of gut environments^7^. Defining how *C. difficile* recruits cooccurring fermentation pathways with systems supporting energy generation and growth has been challenging^8,9^. However, means to definitively track the use of growth-promoting fermentable substrates, and associated recruitment of metabolic pathways, offers opportunities to prevent and treat infections with approaches that need not rely solely on antibiotics.

High-resolution magic angle spinning (HRMAS) nuclear magnetic resonance (NMR) spectroscopy supports studies of real-time metabolism in living cells^10–12^, and is particularly suited to the study of anaerobes as the sealed rotor chamber can maintain an anaerobic environment^12^. HRMAS NMR rotates samples at a ‘magic angle’ of 54.74° relative to the magnetic field during NMR spectrum measurement, improving the sensitivity of signal detection in colloidal or semisolid samples^13^. Detailed studies of metabolism can thus be achieved with a low input biomass of cells^12^. When coupled with cellular metabolism of uniformly carbon-13 (^13^C) labeled substrates, HRMAS NMR’s sensitivity enables definitive tracking of carbon flow through complex metabolic pathways.

Metabolic modeling systems link experimentally obtained substrate and metabolite fluxes to cellular pathways and genes^14–17^. Among modeling approaches, dynamic flux balance analysis (dFBA) simulates time-dependent recruitment of metabolic pathways on an organismal scale given a set of reaction flux constraints and a biological objective such as biomass or adenosine triphosphate (ATP) production^18^. Existing dFBA approaches estimate exchange fluxes from static measurements of the media composition over time^18,19^ for which quantitative platforms, including gas chromatography and mass spectrometry (GC–MS) analyses of *C. difficile-*produced metabolites, have been used^20^. However, the use of NMR to constrain dFBA simulations has been limited by means to translate NMR signals into credible estimated concentrations due to issues in NMR spectral resolution, signal-to-noise levels and susceptibility of ^13^C NMR peaks to amplitude distortions from the nuclear Overhauser effect (NOE)^21^. With means to overcome these limitations, HRMAS NMR offers a promising approach to support dFBA given its nondestructive measurement of isotopic flux in minute quantities of living cells^12,22^.

**Fig. 1:**
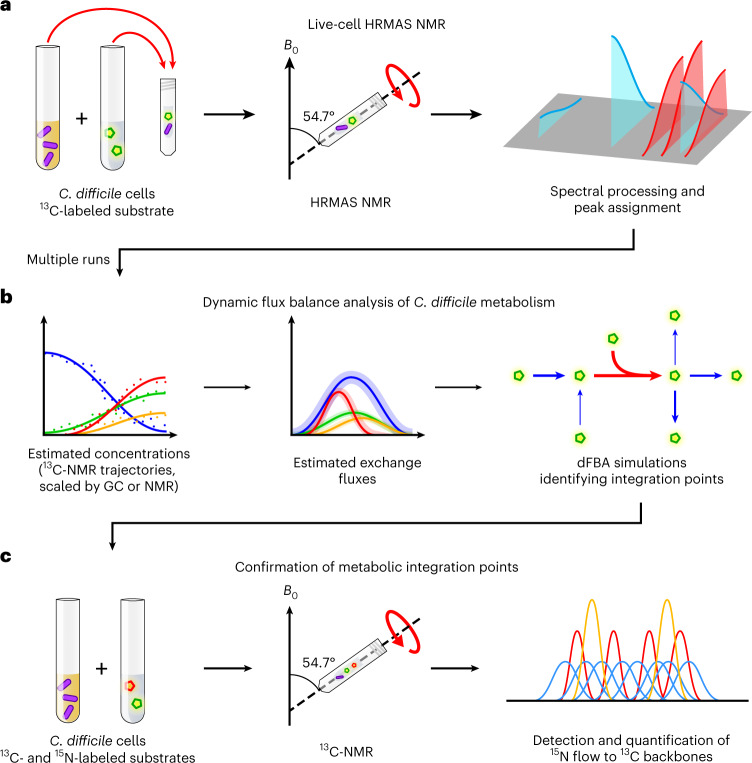
Framework for identifying metabolic integration points using HRMAS NMR with labeled substrates and dFBA. **a**, HRMAS NMR resolves live-cell anaerobic metabolism of ^13^C-labeled substrates. A defined medium containing a ^13^C-labeled substrate is inoculated with *C. difficile* cells in an HRMAS rotor insert. Successive ^1^H- and ^13^C NMR spectra of the growing cells are acquired throughout log-phase growth to monitor metabolism of the labeled substrate. NMR spectra are processed, and peaks are assigned to metabolites using ^1^H-^13^C HSQC spectra and reference data. **b**, NMR signal trajectories inform dFBA simulations to identify metabolic integration points. Logistic curves for metabolites are fit to integrated ^13^C-signal trajectories and scaled to estimated concentrations using information from standard solutions measured by GC or NMR. Estimated metabolite exchange fluxes are derived from the logistic curves representing multiple NMR runs to constrain dFBA simulations. Metabolic integration points are identified where dFBA solutions predict substrates to exchange electrons or functional groups. **c**, ^13^C NMR of ^13^C- and ^15^N-labeled substrates confirms dFBA predictions of nutrient flow. *C. difficile* cells are grown in defined media containing ^13^C- and ^15^N-labeled substrates under NMR acquisition. ^15^N flow to ^13^C backbones is measured by quantifying the relative areas of split ^13^C NMR subpeaks at the ^13^C-alanine alpha carbon.

We leveraged HRMAS ^13^C NMR to constrain dFBA within genome-scale metabolic models to define complex dynamics in *C. difficile*’s metabolism. HRMAS NMR spectra were acquired from living *C. difficile* cells fermenting defined ^13^C-labeled substrates over time (Fig. 1a). The ^13^C NMR integrated signal curves were then normalized using experimental standards to provide constraints for dFBA simulations (Methods, Estimation of exchange fluxes for dFBA and Fig. 1b). Analyses revealed alanine production as a central metabolic integration point in amino acid-fermenting *Clostridia* to support cooccurring oxidative and reductive reactions across amino acid and glycolytic metabolism. The confirmation of model predictions leveraged a generalizable NMR approach to simultaneously track carbon and nitrogen flow through cellular metabolism, using amplification of signal from weak NMR-active nuclei, such as nitrogen-15 (^15^N), through strong NMR-active nuclei, such as ^13^C (Fig. 1c).

## Results

### HRMAS ^13^C NMR elucidates *C. difficile*’s complex metabolism

**Extended Data Fig. 1:**
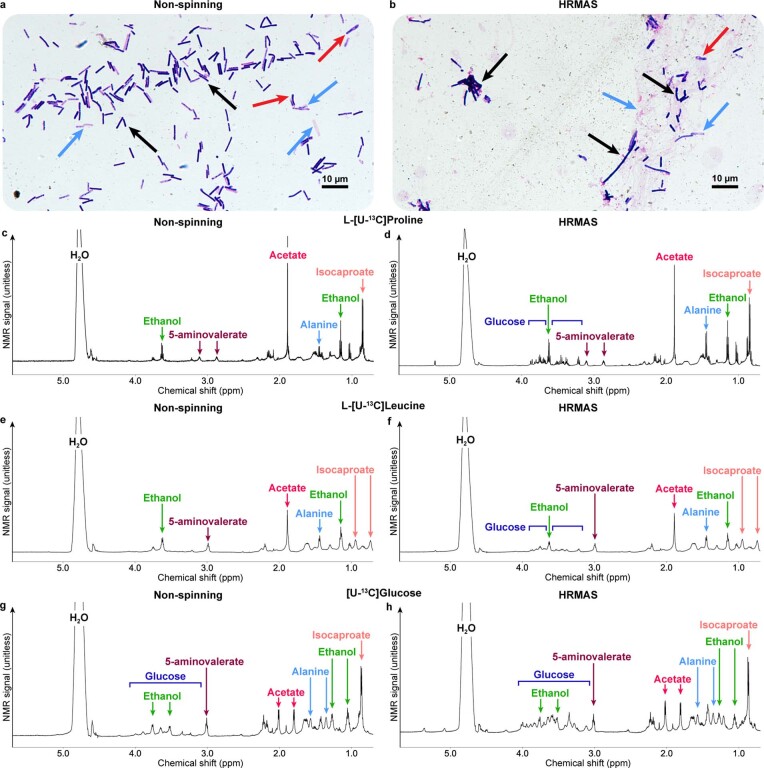
Metabolic consistency of HRMAS acquisition with standard anaerobic cultures in Kel-F rotor inserts. Analyses of *C. difficile* cultures grown in MMM in a sealed Kel-F rotor insert, either incubated for 36 h at 37 °C without spinning (“nonspinning”) or subjected to the HRMAS radiofrequency pulse and spinning program for 36 h (“HRMAS”). (**a–b**) Optical microscopy images of Gram-stained cultures grown in the (**a**) non-spinning or (**b**) spinning condition; each microscopy image represents three biological replicates yielding similar results. Colored arrows indicate vegetative cells (black arrows), lysed cells and debris (blue arrows), and sporulating cells (red arrows). (**c–d**) ^1^H-NMR spectra of cultures grown in MMM with [U-^13^C]proline in the (**c**) non-spinning or (**d**) HRMAS condition. Axes: unitless NMR signal (*y* axis) versus chemical shift in ppm (*x* axis). Labels identify ^1^H peaks attributed to selected metabolites. (**e–f**) Cultures grown in MMM with [U-^13^C]leucine in the (**e**) non-spinning or (**f**) HRMAS condition; axes and labels as in (**c**–**d**). (**g–h**) Cultures grown in MMM with [U-^13^C]glucose in the (**g**) non-spinning or (**h**) HRMAS condition; axes and labels as in (**c**–**d**).

To investigate the progression of co-occurring amino acid and glycolytic fermentations in *C. difficile*, we measured HRMAS NMR time series of proton (^1^H) and ^13^C NMR spectra from living *C. difficile* cells. Cultures were grown in modified minimal medium (MMM) that replaced a given carbon source at natural isotope abundance with its uniformly labeled carbon-13 isotopologue: l-[U-^13^C]proline, l-[U-^13^C]leucine or [U-^13^C]glucose (Fig. 1a). *C. difficile* cultures grown under the conditions of HRMAS acquisition demonstrated consistent metabolic findings with conventional anaerobic cultures (Extended Data Fig. 1, Supplementary Table 1 and Methods, Nonspinning control experiments).

**Fig. 2:**
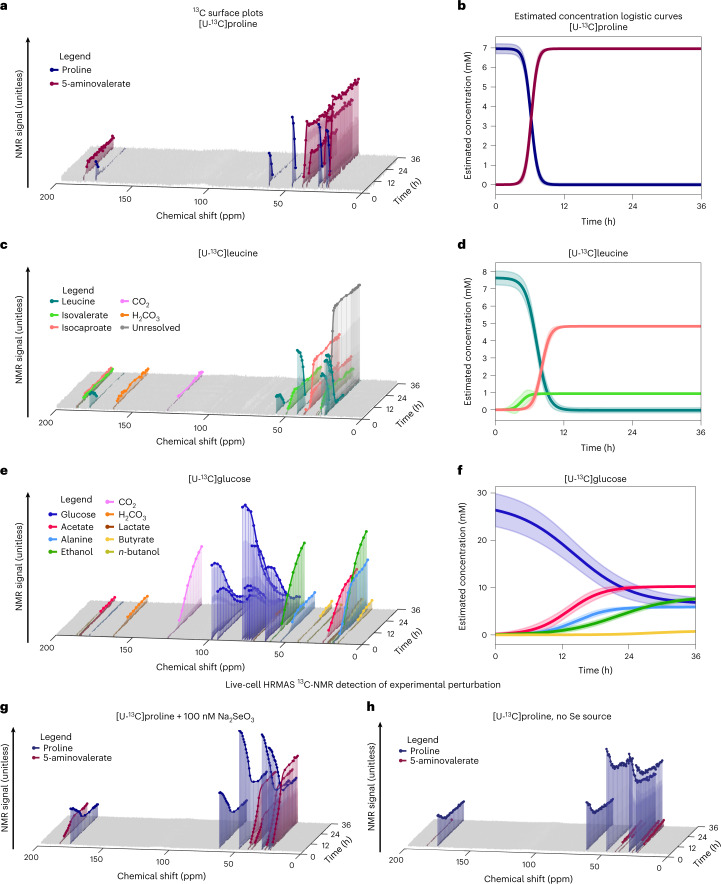
HRMAS ^13^C NMR of *C. difficile* grown with U-^13^C fermentable substrates. **a**, HRMAS NMR stack plot of growth in MMM with 7.0 mM [U-^13^C]proline. The legend shows color-coding of input proline (dark blue) and 5-aminovalerate (dark red). The *x* axis shows ^13^C NMR chemical shift (ppm), the *y* axis shows time (h) and *z* axis shows NMR signal (unitless). **b**, Logistic plots depicting estimated concentration (mM) of [U-^13^C]proline and [U-^13^C]5-aminovalerate versus time (h). Trajectories are calculated and normalized as described in the Methods (section Aligning the time axes and calculating average logistic coefficients). Bold lines depict mean trajectories across three experimental replicates; shaded regions depict the 95% confidence interval of the mean. **c**, HRMAS NMR stack plot of growth in MMM with 7.6 mM [U-^13^C]leucine. The legend shows carbons in leucine (blue-green) and detected metabolites; axes as in **a**. The 25 ppm peaks (gray) of isovalerate and isocaproate could not be resolved due to extensive overlap. **d**, Logistic plots depicting estimated concentration of [U-^13^C]leucine and detected metabolites versus time. Axes and curves as in **b**. **e**, HRMAS NMR stack plot of growth in MMM with 27.5 mM [U-^13^C]glucose. The legend shows color-coding of carbons in glucose (blue) and metabolites appearing over 36 h of cellular metabolism; axes as in **a**. **f**, Logistic plots depicting estimated concentration of [U-^13^C]glucose and detected metabolites versus time. Axes and curves as in **b**. **g**,**h**, ^13^C NMR time series of *C. difficile* cultures in MMM with 30 mM [U-^13^C]proline with (**g**) or without (**h**) 100 µM sodium selenite. Legend shows color-coding of input proline (dark blue) and 5-aminovalerate (dark red); axes as in **a**.

**Extended Data Fig. 2:**
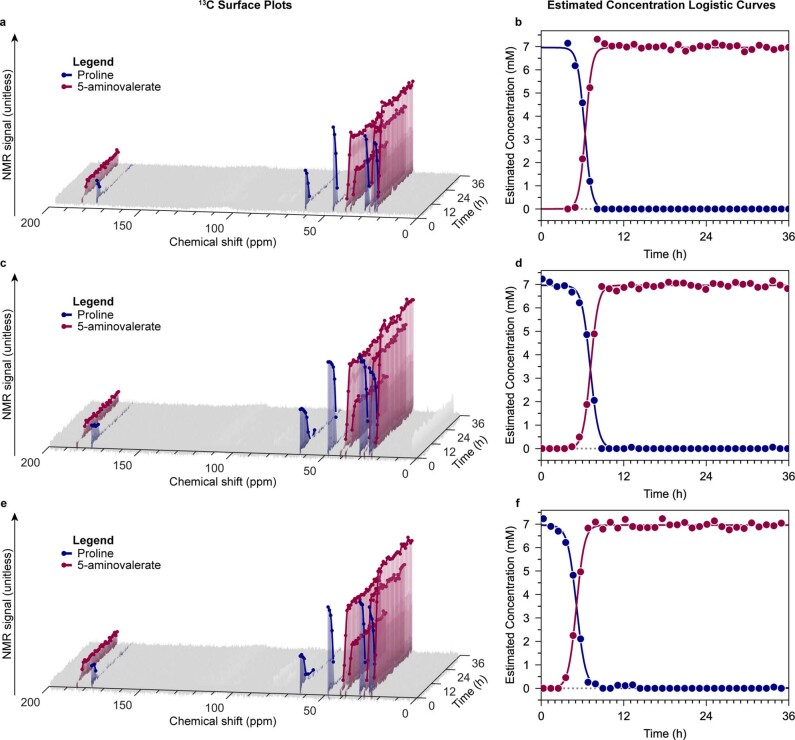
HRMAS ^13^C NMR of *C. difficile* grown with L-[U-^13^C]proline. (**a**) Surface plot of ^13^C-NMR time series for experimental replicate 1 with trajectories of peaks attributed to proline (dark blue) and 5-aminovalerate (dark red). Axes: unitless NMR signal (*z* axis) versus chemical shift in ppm (*x* axis) versus time in hours (*y* axis). (**b**) Scatter plot for experimental replicate 1 of estimated metabolite concentrations (mM) with logistic curve fit versus time in hours. (**c**) Surface plot of ^13^C-NMR time series for experimental replicate 2. Lines and axes as in (**a**). (**d**) Scatter plot for experimental replicate 2 of estimated metabolite concentrations, axes as in (**b**). (**e**) Surface plot of ^13^C-NMR time series for experimental replicate 3. Lines and axes as in (**a**). (**f**) Scatter plot for experimental replicate 3 of estimated metabolite concentrations, axes as in (**b**).

As a Stickland amino acid-fermenting *Clostridium*^6^*, C. difficile* preferentially metabolizes amino acids, such as proline and leucine, with simple sugars, such as glucose^23^. These substrates are known to drive rapid pathogen growth in vivo^9^, a prerequisite for the development of symptomatic disease^23,24^. *C. difficile*’s proline reductase reduces proline to a single metabolite, 5-aminovalerate^4,25^. Biochemical and protein interaction studies have identified coupling of proline reductase activity to proton ejection by the bacterial Rnf complex, which supports ATP synthesis^26,27^. HRMAS NMR of *C. difficile* cultures growing in media containing l-[U-^13^C]proline revealed complete proline consumption within 5 hours (h), producing [U-^13^C]5-aminovalerate (Fig. 2a,b, Extended Data Fig. 2 and Supplementary Fig. 1).

**Extended Data Fig. 3:**
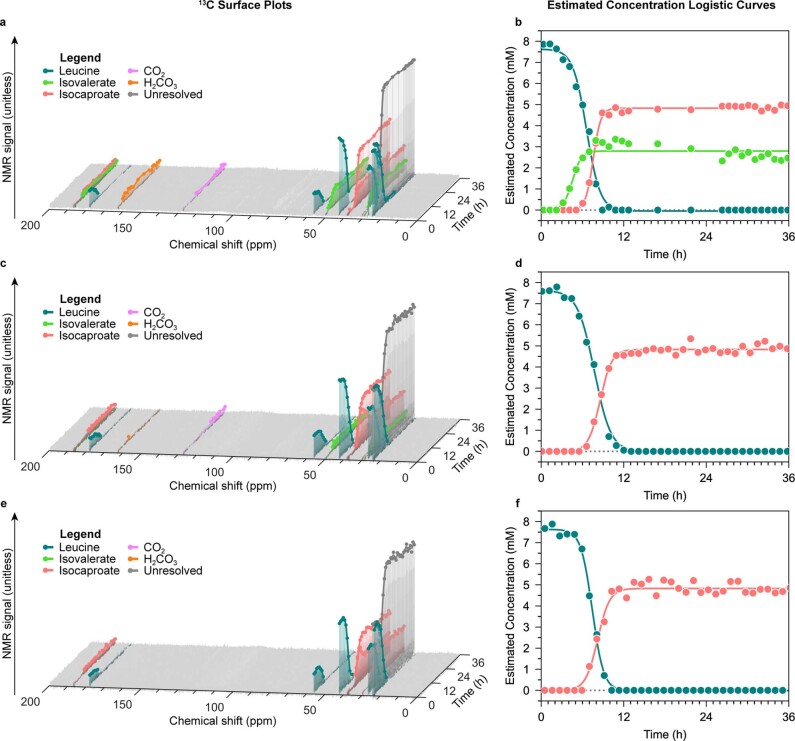
HRMAS ^13^C NMR of *C. difficile* grown with L-[U-^13^C]leucine. (**a**) Surface plot of ^13^C-NMR time series for experimental replicate 1 with trajectories of peaks attributed to leucine (teal) and its fermentation products. Axes: unitless NMR signal (*z* axis) versus chemical shift in ppm (*x* axis) versus time in hours (*y* axis). (**b**) Scatter plot for experimental replicate 1 of estimated metabolite concentrations (mM) with logistic curve fit versus time in hours. (**c**) Surface plot of ^13^C-NMR time series for experimental replicate 2. Lines and axes as in (**a**). (**d**) Scatter plot for experimental replicate 2 of estimated metabolite concentrations, axes as in (**b**). **[U-**^**13**^**C]**Isovalerate in this run lacked sufficient signal to infer a credible trajectory. (**e**) Surface plot of ^13^C-NMR time series for experimental replicate 3. Lines and axes as in (**a**). (**f**) Scatter plot for experimental replicate 3 of estimated metabolite concentrations, axes as in (**b**).

In contrast, *C. difficile* ferments l-leucine through separate oxidative and reductive pathways^5,24^. Oxidative fermentation of leucine to isovalerate and CO_2_ produces two reducing equivalents of ferredoxin and one equivalent of ATP, whereas the reductive leucine pathway yields isocaproate and consumes four net reducing equivalents^5,24,28^. HRMAS NMR revealed complete metabolism of l-[U-^13^C]leucine within 13 h, with levels of [^13^C]isovalerate and [^13^C]isocaproate rising over 2.7–8.7 and 7.4–13.0 h, respectively (Fig. 2c,d, Extended Data Fig. 3 and Supplementary Fig. 2).

**Extended Data Fig. 4:**
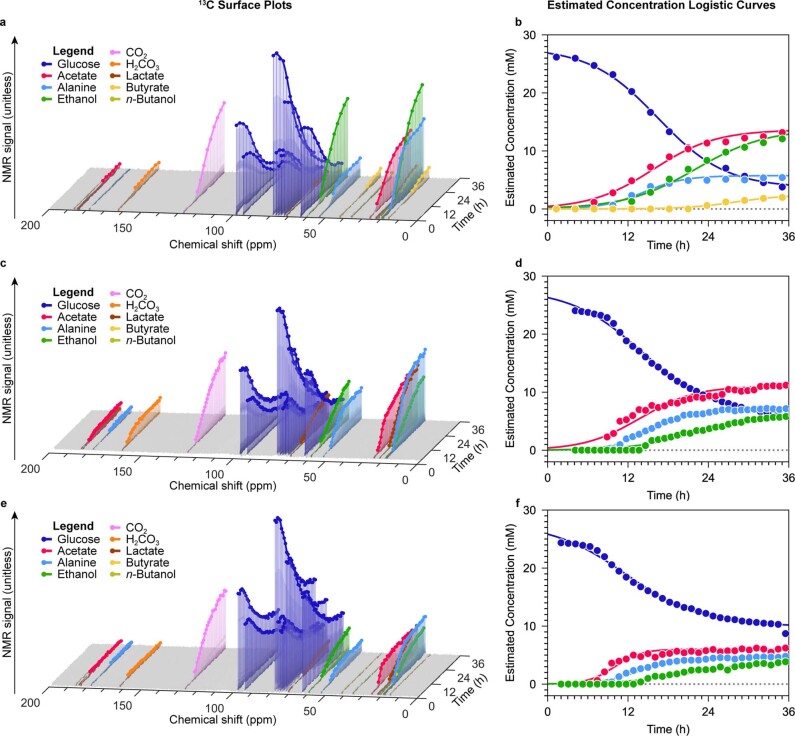
HRMAS ^13^C NMR of *C. difficile* grown with [U-^13^C]glucose. (**a**) Surface plot of ^13^C-NMR time series for experimental replicate 1 with trajectories of peaks attributed to glucose (blue) and its fermentation products. Axes: unitless NMR signal (*z* axis) versus chemical shift in ppm (*x* axis) versus time in hours (*y* axis). (**b**) Scatter plot for experimental replicate 1 of estimated metabolite concentrations (mM) with logistic curve fit versus time in hours. (**c**) Surface plot of ^13^C-NMR time series for experimental replicate 2. Lines and axes as in (**a**). (**d**) Scatter plot for experimental replicate 2 of estimated metabolite concentrations, axes as in (**b**). (**e**) Surface plot of ^13^C-NMR time series for experimental replicate 3. Lines and axes as in (**a**). (**f**) Scatter plot for experimental replicate 3 of estimated metabolite concentrations, axes as in (**b**).

*C. difficile* ferments glucose through progressive oxidative and reductive pathways that support the organism’s energetic and metabolic needs. The highest flux oxidative pathways convert glycolytic pyruvate to acetate, with production of ATP and further extraction of electrons that can feed into the Rnf system. Corresponding high-flux reductive pathways produce ethanol, lactate, butyrate and butanol with regeneration of electron carriers, as well as reactions, including electron bifurcating systems, that extract further energy to support energetic needs^6,28^. HRMAS NMR of *C. difficile* grown with [U-^13^C]glucose identified [^13^C]acetate at 7 h and [^13^C]alanine at 10 h (Fig. 2e,f). Metabolic products of reductive glucose metabolism were detected by 13 h with production of [^13^C]ethanol, followed by [^13^C]lactate at 21 h, [^13^C]butyrate at 24 h and *n*-[^13^C]butanol at 35 h (Fig. 2e,f, Extended Data Fig. 4 and Supplementary Fig. 3).

To assess the responsiveness of live-cell HRMAS NMR to metabolic perturbations, we next compared *C. difficile*’s proline reductase metabolism, a selenium-dependent pathway^25,29^, in the presence of an excess of 30 mM [U-^13^C]proline. Compared to *C. difficile* grown in MMM with 100 µM sodium selenite, cells grown in selenium-deficient MMM produced only 7% as much [U-^13^C]5-aminovalerate signal (compare Fig. 2g with h).

### Using NMR data to constrain metabolic simulations

We next estimated metabolite exchange fluxes from the HRMAS ^13^C NMR trajectories, linking NMR datasets to the supporting metabolic pathways, genes and their dynamic recruitment over the experimental time series (Fig. 1b). To estimate metabolite concentration trajectories, the integrated NMR signal over time for each ^13^C compound was first fit to a logistic curve, using the equation:

1 $$\begin{array}{*{20}{c}} {f\left( x \right) = \frac{L}{{1 + {\mathrm{e}}^{ - k\left( {x - x_0} \right)}}} + C} \end{array}$$

where *L* is the upper asymptote, *k* is the growth rate and *x*_0_ is the inflection point of the sigmoidal curve (Supplementary Table 2). For the U-^13^C substrates, the constant *C* accounts for remaining substrate after 36 h; for the products, *C* equals zero. Final concentrations of ^13^C-labeled products were estimated using the known U-^13^C substrate concentration, ratios of ^13^C NMR peak area and empirically determined correction factors for NOE-induced signal amplification (Estimation of exchange fluxes for dFBA). Logistic equation (1) was scaled using estimated concentrations across replicate runs, and differentiated to yield exchange flux constraints for dFBA simulations (Fig. 2b,d,f and Supplementary Table 3):

2 $$\begin{array}{*{20}{c}} {f\,^{\prime} \left( x \right) = \frac{{kL{\mathrm{e}}^{ - k\left( {x - x_0} \right)}}}{{\left( {1 + {\mathrm{e}}^{ - k\left( {x - x_0} \right)}} \right)^2}}} \end{array}$$

### dFBA predicts rapid ATP generation by Stickland metabolism

dFBA simulations used icdf843, an updated metabolic model for *C. difficile* (Metabolic modeling and Supplementary Table 4). Model exchange fluxes for NMR-detected metabolites were constrained to the 95% confidence bounds of the logistic derivative equation (2) evaluated over a simulated 36-h time series (Metabolic modeling and Supplementary Table 5). Solutions used ATP hydrolysis as the biological objective to provide consistency across phases of growth and sporulation (Supplementary Table 6). Flux variability analysis (FVA) solutions, computed at each simulated timepoint, estimated flux bounds for reactions accommodating an objective flux within 0.5% of the optimal value (Supplementary Tables 7 and 8).

**Fig. 3:**
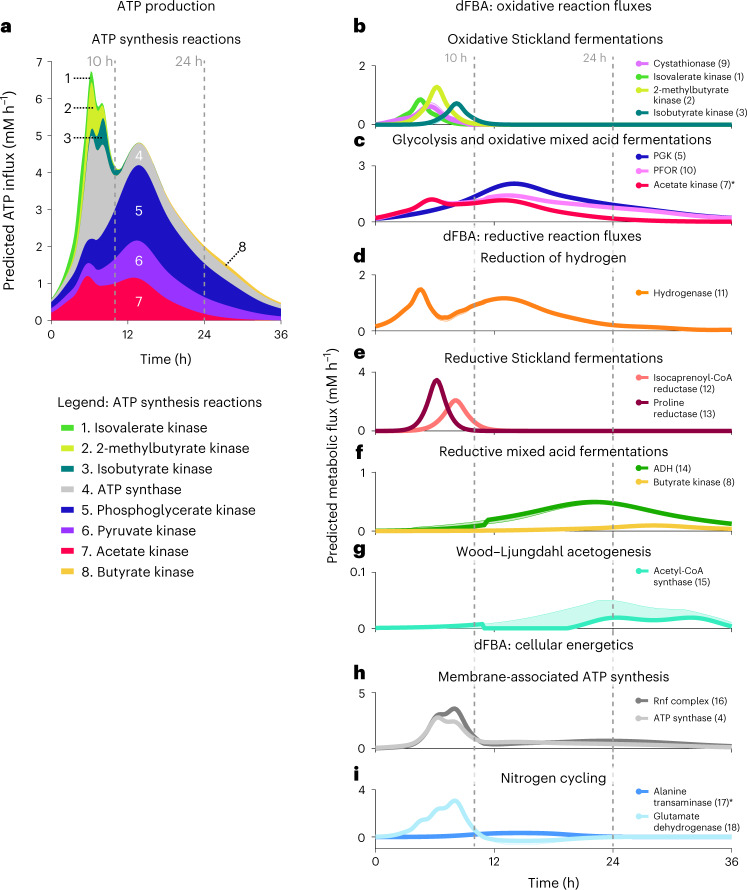
dFBA-predicted reaction fluxes and production of key metabolic intermediates linking glycolysis and Stickland metabolism. **a**–**i**, dFBA-predicted reaction fluxes over 36 h of metabolism (*x* axis). The *y* axis shows inferred flux in mM h^−1^. **a**, Inferred flux of reactions contributing to the production of ATP versus time, including isovalerate kinase (light green, 1), 2-methylbutyrate kinase (yellow-green, 2), isobutyrate kinase (dark green, 3), ATP synthase (light gray, 4), PGK (dark blue, 5), pyruvate kinase (purple, 6), acetate kinase (red, 7) and butyrate kinase (yellow, 8). **b**–**i**, The model-inferred recruitment of oxidative and reductive pathways, including metabolic integration between amino acid and glycolytic metabolism. Bold lines depict FBA-predicted optimal metabolic fluxes; shaded regions depict FVA-predicted flux tolerances supporting objective flux within 0.5% of the optimal solution. **b**, Estimated flux of oxidative Stickland fermentations represented by cystathionase (purple, 9), isovalerate kinase (light green, 1), 2-methylbutyrate kinase (yellow-green, 2) and isobutyrate kinase (dark green, 3). **c**, Estimated glycolytic flux represented by PGK (dark blue, 5) and oxidative mixed-acid fermentations represented by pyruvate:ferredoxin oxidoreductase (PFOR; pink, 10) and acetate kinase (red, 7; *shaded FVA upper bound (1,000 mM h^−1^) is not displayed because it exceeds the axes limits). **d**, Reductive hydrogen production by iron hydrogenase (orange, 11). **e**, Reductive Stickland fermentation represented by isocaprenoyl-CoA reductase (light red, 12) and proline reductase (dark red, 13). **f**, Reductive mixed-acid fermentations represented by ethanol dehydrogenase (ADH; green, 14) and butyrate kinase (yellow, 8). **g**, Wood–Ljungdahl acetogenesis represented by acetyl-CoA synthase (blue-green, 15). **h**, Membrane-associated ATP synthesis represented by ATP synthase (light gray, 4) and the gradient-producing Rnf complex (medium gray, 16). **i**, Nitrogen cycling via alanine transaminase (medium blue, 17, *shaded FVA upper and lower bounds (± 1,000 mM h^−1^) are not displayed because they exceed the axes limits) and glutamate dehydrogenase (light blue, 18). Positive glutamate dehydrogenase flux depicts the forward reaction oxidizing glutamate to 2-oxoglutarate; negative flux, below the *x* axis, depicts the reverse reaction.

**Fig. 4:**
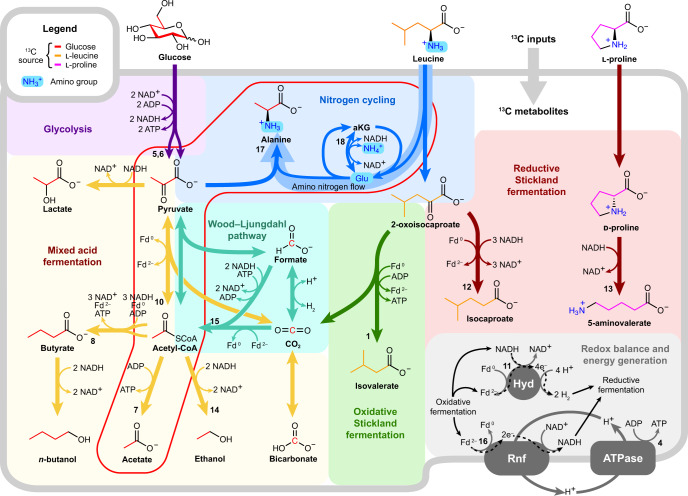
HRMAS NMR and dFBA-informed metabolic map for *C. difficile*. Diagram shows an updated metabolic map for *C. difficile* highlighting alanine’s role in nitrogen cycling (blue) and integration of glycolytic and mixed-acid fermentations (yellow) with Stickland oxidative (green) and reductive (dark red) reactions that release abundant amino nitrogen. The map also includes the Wood–Ljungdahl pathway (blue-green) and iron hydrogenase (‘Hyd’), predicted by dFBA to serve as key electron sinks and the Rnf-ATPase system responsible for ATP-producing electron transfer between oxidative and reductive fermentations (gray). Numbered reactions correspond to the reaction legend in Fig. 3 and Supplementary Table 10. The red-lined box indicates central reactions linking pyruvate, acetate and alanine across glycolytic and Stickland fermentations.

dFBA simulations predicted three stages of metabolism with progressive recruitment of oxidative reactions driving ATP synthesis (Fig. 3a) and co-occurring reductive reactions to accept electrons and sustain oxidative flux. Model simulations predicted 36.1% of overall ATP synthesis during the first 10 h of metabolism with peak production occurring at 6.2 h. Metabolism during this period was marked by the rapid oxidative fermentation of leucine (Figs. 3a,b and 4, reaction 1) with progressive recruitment of oxidative Stickland amino acid fermentation pathways for isoleucine (Fig. 3a,b, reaction 2), valine (Fig. 3a,b, reaction 3) and cysteine (Fig. 3b, reaction 9). The substrate-level phosphorylation reactions from oxidative amino acid metabolism were estimated to comprise 27.6% of ATP production during this interval. Simulations predicted an additional 38.9% of ATP production as oxidative glycolytic (Figs. 3a,c and 4, reactions 5 and 6) and mixed-acid fermentation reactions were recruited (Figs. 3a,c and 4, reaction 7). dFBA solutions predicted progressive recruitment of reductive pathways during this period supporting the combined oxidative fermentations, starting with the evolution of molecular hydrogen from *C. difficile*’s hydrogenase system (Figs. 3d and 4, reaction 11), followed by Stickland reductive reactions for leucine and proline (Figs. 3e and 4, reactions 12 and 13).

Simulations estimated 49.8% of overall ATP synthesis to occur over 10–23 h of metabolism, of which 84.1% was predicted to originate from glycolytic and mixed-acid fermentations, peaking at 14 h. After the depletion of leucine and proline pools for reductive metabolism, model simulations predicted recruitment of solvogenic ethanol fermentation (Figs. 3f and 4, reaction 14) and continued hydrogenase flux as the primary electron sinks.

Late-stage metabolism, occurring over 24–36 h, was predicted to account for the remaining 14.1% of overall ATP synthesis, marked by a shift to reductive, solvogenic reactions producing butyrate and *n-*butanol (Figs. 3a,f and 4, reaction 8), along with Wood–Ljungdahl acetogenesis (Figs. 3g and 4, reaction 15). Substrate-level phosphorylation in butyrate fermentation contributed an estimated 6.1% of ATP synthesis during this interval, whereas residual oxidative glucose fermentation was predicted to sustain 71.3% of ATP synthesis during this time.

Throughout all three stages of metabolism, dFBA simulations predicted that the Rnf complex (Figs. 3h and 4, reaction 14 and redox balance and energy generation) harnessed the energy cascade between oxidative and reductive metabolism to generate proton gradients and ATP production through *C. difficile*’s F-type ATP synthase (Figs. 3a,h and 4, reaction 7). Model solutions estimated a peak in membrane-associated ATP synthesis at 6.4 h, concurrent with the maximum reductive flux from amino acid fermentations. Thereafter, simulations predicted ATP generation to be sustained primarily via electron transfer from mixed-acid fermentation reactions to the Rnf complex and *C. difficile*’s F-type ATP synthase (Figs. 3h and 4, reaction 7).

### dFBA predicts glycolysis-Stickland intergration at alanine

dFBA solutions, informed by HRMAS ^13^C NMR trajectories, predicted the transamination of pyruvate to alanine (Figs. 3i and 4, reaction 15) as a central integration point for glycolytic and Stickland amino acid metabolism to support energy generation, nitrogen handling and cellular growth (Fig. 4, red-lined box). Model solutions predicted net oxidative deamination of amino acids over the first 10 h of metabolism (Figs. 3i and 4, reaction 16), particularly from the rapid fermentation of leucine. By 11 h, with the progressive recruitment of high-flux glycolytic metabolism, solutions predicted the transamination of pyruvate to alanine, with leucine-origin ammonia flowing through glutamate to supply 56.4% of alanine’s amino nitrogen (Fig. 4, amino nitrogen flow and Supplementary Table 6).

### Alternate dFBA objectives enhance model predictions

To evaluate the consistency of model predictions for timed metabolic progression and associated points of pathway integration, we performed dFBA and FVA simulations using alternate model objectives of biomass production and exopolysaccharide synthesis (Supplementary Fig. 4). Notably, the inferred pathway fluxes remained consistent across objectives with modest differences in flux magnitudes and timing for some reactions. In the case of cysteine fermentation (Supplementary Fig. 4b,k,t, reaction 9), cystathionase activity was predicted to supply central carbon pools during early metabolism under ATP hydrolysis. In constrast, maximum cystathionase flux occured during late metabolism when using biomass as the objective. The greatest difference occurred when using exopolysaccharide as the objective where flux occurred consistently throughout the time series, with a maximum during the first 10 h of metabolism, per shunting of produced pyruvate into gluconeogenesis reactions to generate glucose for expolysaccharide biosynthesis.

Additional nuances in inferred reaction fluxes among the model objectives included increased glycolytic flux in the biomass simulation and decreased glycolytic flux in the exopolysaccharide simulation versus the ATP hydrolysis condition, as represented by phosphoglycerate kinase (PGK) (Supplementary Fig. 4c,l,u, reaction 5). The negative PGK flux around 6 h, under the exopolysaccharide objective, inferred diversion of products via the reverse reaction into gluconeogenic pathways to supply the exopolysaccharide synthesis pathway with glucose residues.

Aside from the aforementioned differences, the inferred flux trajectories remained similar to the ATP hydrolysis objective. All three objectives predicted the timed progression from amino acid to glycolytic fermentations, and alanine’s biosynthesis as a metabolic integration point supporting the transfer of amino groups from deaminating Stickland reactions to glycolytic pyruvate.

### Concurrent NMR tracking of ^15^N and ^13^C through metabolism

To confirm model predictions of amino nitrogen flow from fermented leucine to glycolytic pyruvate, we developed an NMR approach to simultaneously track cellular carbon and nitrogen flow. Tracking of less sensitive NMR-active nuclei, such as ^15^N, through cellular metabolism has been more challenging than that of more sensitive NMR-active nuclei such as ^13^C and ^1^H, as ^15^N produces 15-fold less signal than ^13^C^30^. However, NMR J-coupling between ^13^C and covalently bound ^15^N induces predictable patterns of nuclear spin–spin splitting and isotope-effect shifting in ^13^C signals^31–33^, enabling detection of the less sensitive ^15^N nucleus in the more sensitive ^13^C NMR spectrum (Analyses of ^15^N-^13^C peak splitting).

To confirm the feasibility of amplifying signal from less sensitive NMR nuclei in the spectrum of more sensitive NMR nuclei, we first evaluated ^13^C-induced splitting of acetate’s peaks in the ^1^H spectrum of cells grown with [U-^13^C]glucose. Signal from the methyl hydrogens of [^13^C_2_]acetate was split into double-doublet peaks, indicating well-resolved ^1^H spin coupling with the ^13^C nucleus in the methyl group (*J* of roughly 34 Hz) and long-range coupling with the ^13^C nucleus in the carboxyl group (*J* of roughly 53 Hz; Extended Data Fig. 6b). Moreover, the amplification of ^13^C signal in the ^1^H spectrum showed 30 times higher signal-to-noise ratio (S/N) than in the ^13^C spectrum alone (Extended Data Fig. 6b), demonstrating that spin–spin splitting in a more sensitive NMR spectrum (^1^H) offers enhanced detection of comparatively less sensitive NMR nuclei (^13^C).

Leveraging this property of NMR physics, we tracked simultaneous flow of ^13^C backbones from [U-^13^C]glucose and ^15^N amino groups from [^15^N]leucine to form [^13^C,^15^N]alanine (Figs. 1c and 4, nitrogen cycling).

### NMR-detected ^15^N-^13^C bonding confirms metabolic integration

**Fig. 5:**
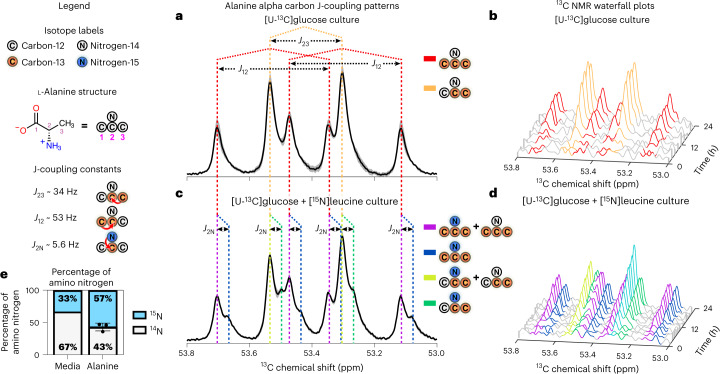
^13^C NMR detects ^15^N amino nitrogen flow from Stickland-fermented leucine to glycolytic ^13^C backbones in alanine. **a**, ^13^C NMR spectra for alanine’s alpha carbon (53.0–53.8 ppm) after growth in MMM media with 27.5 mM [U-^13^C]glucose and 7.6 mM natural-abundance leucine for 36 h. Bold black line with gray shading depicts the mean ^13^C NMR signal ± s.e.m. across three biological replicates. Red-dotted lines indicate J-coupled peaks associated with [U-^13^C]alanine. Orange-dotted lines indicate J-coupled peaks associated with [2,3-^13^C]alanine resulting from ^12^CO_2_ assimilation with [U-^13^C]acetate, via pyruvate:ferredoxin oxidoreductase. **b**, Time series of J-coupled peaks at alanine’s alpha carbon over 24 h of metabolism, color coded as shown in **b**. **c**, ^13^C NMR spectra after growth in 27.5 mM [U-^13^C]glucose and 7.6 mM [^15^N]leucine showing J-coupled split peaks from [U-^13^C,^15^N] alanine (blue) or [2,3-^13^C_,_^15^N] alanine (green). Purple lines show peaks with a mixture of [U-^13^C,^15^N]alanine and [U-^13^C, ^14^N]alanine; yellow-green lines show peaks with [2,3-^13^C_,_^15^N]alanine and [2,3-^13^C_,_^14^N]alanine. ^13^C NMR signal curve displays center and variability as in **a**. **d**, Time series of J-coupled peaks, color coded as shown in **c**. * The asterisk over the aqua-colored peak indicates combined [2,3-^13^C,^14^N]alanine, [2,3-^13^C,^15^N]alanine and [U-^13^C, ^15^N]alanine. **e**, Table shows percentages of ^14^N and ^15^N amino nitrogen in starting media with [U-^13^C]glucose and [^15^N]leucine, and resulting integrated ^14^N-^13^C and ^15^N-^13^C percentages (± s.d.) on alanine after 36 h of growth (Supplementary Table 9). *N* = 3 biological replicates to assess variability in the ^15^N:^14^N isotope ratio.

**Extended Data Fig. 5:**
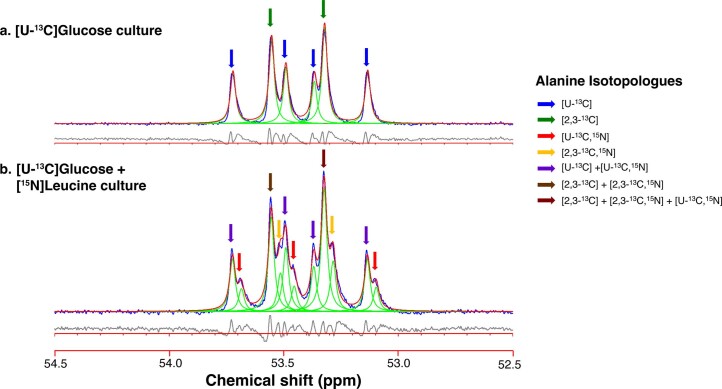
Curve-fitted ^13^C NMR spectral region of alanine’s α-carbon following the growth of live *C. difficile* in [U-^13^C]glucose with natural abundance leucine or [^15^N]leucine. (**a**) Spectrum of culture grown in media containing natural abundance leucine. (**b**) Spectrum of culture grown in media containing [^15^N]leucine. In each resonance spectrum, blue indicates the curve of the experimental raw data; red indicates the overall fitted curve; and green indicates the individual fitted peaks; the grey line indicates the difference between the experimental and fitted curves.

HRMAS ^13^C NMR time series of *C. difficile* grown with [U-^13^C]glucose and natural-abundance leucine revealed [2,3-^13^C]alanine and [U-^13^C]alanine in a 1:1 ratio (Fig. 5a,b, Extended Data Fig. 5a and Supplementary Table 9), indicating substantial assimilation of ^12^CO_2_ with [U-^13^C]acetate, an activity reported to occur via pyruvate:ferredoxin oxidoreductase in many species of *Clostridia*^34^. *C. difficile* grown in the presence of [U-^13^C]glucose and [^15^N]leucine showed ^15^N-induced splitting (*J* ≅ 5.6 Hz) and isotope-effect shifting (*δ* ≅ 0.025 parts per million (ppm)) of the ^13^C peaks associated with alanine’s alpha carbon and mixed populations of [^15^N]alanine and [^14^N]alanine (Fig. 5c,d, Extended Data Fig. 5b and Supplementary Table 9). Although [^15^N]leucine represented only 33% of amino-group nitrogen in the starting media, 57% (standard deviation (s.d.), 4%) of [^13^C]alanine carried the ^15^N isotope (Fig. 5e), confirming enriched transfer of the ^15^N amino group from fermented [^15^N]leucine to [^13^C]alanine.

## Discussion

We illustrate advancements in HRMAS NMR and genome-scale metabolic modeling to track single carbon and nitrogen flow through complex anaerobe metabolism. The sealed rotor chamber used in HRMAS NMR maintains an anaerobic environment, ensuring maintenance of reducing conditions, conservation of mass and biological containment. The nondestructive nature of HRMAS NMR spectroscopy enables high-resolution analyses of living cells in small reaction volumes, on the order of tens of microliters, whereas alternatives using GC, MS or regular solution NMR often require orders of magnitude more volume and cellular mass to support longitudinal analyses. The acquisition of HRMAS NMR datasets is also far more rapid and does not require further extraction or preparation. Additionally, we show that HRMAS NMR detects metabolic shifts accompanying defined perturbations in nutrient availability as demonstrated by the impact of selenium limitation on *C. difficile*’s metabolism of [U-^13^C]proline by its proline reductase selenoenzyme^25^.

Longitudinal tracking of fermentable ^13^C-labeled substrates supports high-resolution monitoring of individual pathways within complex nutrient conditions. The cellular scope of genome-scale metabolic models provides means to incorporate multiple NMR datasets tracking fermentable substrates into a unified dFBA solution with inference of flux through pathways not directly captured by NMR analyses and of cellular or experimental objectives that may include ATP generation, biomass or production of industrially and physiologically important metabolites. To overcome previous limitations in using NMR time series data for genome-scale predictions of cellular metabolism, we present approaches to transform integrated ^13^C NMR trajectories into flux estimates to support dFBA simulations (Estimation of exchange fluxes for dFBA). The approaches accommodate linear as well as complex branching pathways, such as for proline and glucose fermentation, respectively.

Our use of ^15^N-^13^C J-coupling to amplify less NMR-sensitive ^15^N nuclei with more sensitive ^13^C NMR presents a conceptual foundation for the simultaneous tracking of atomic species to determine their chemical and biological relevance. ^13^C NMR time series quantified enriched ^15^N flow from fermented [^15^N]leucine to glucose-origin ^13^C backbones, confirming alanine formation as a central integration point between glycolytic and Stickland fermentations. This use of NMR J-coupling may be extended to covalent bonding with other NMR-active nuclei such as ^1^H or ^17^O to track distinct spin-active nuclei in an NMR time series and experimentally confirm metabolic integration points predicted by dFBA (Analyses of ^15^N-^13^C peak splitting).

Our findings inform in vivo behaviors of *C. difficile* by illustrating how the pathogen rapidly recruits amino acid fermentation pathways when presented with abundant fermentable amino acids and carbohydrates, conditions that occur in vivo after antibiotic ablation of the microbiota^9^. NMR-informed, dFBA solutions indicated early recruitment of Stickland oxidative and reductive pathways to drive rapid ATP generation via substrate-level phosphorylation reactions and via *C. difficile*’s membrane-associated ATP synthase, whereas glycolytic and mixed-acid fermentation pathways were predicted to provide metabolic support as pools of fermentable amino acids were depleted. The consistency of dFBA solutions among differing model objectives demonstrates the use of this approach to thoroughly interrogate complex microbial metabolism and enhance the robustness of predictions, as well as to identify specific nuances in metabolic and cellular behaviors that may occur when optimizing for a particular objective.

NMR time series and genome-scale metabolic analyses also identified a unique strategy Stickland fermenters use to integrate Stickland metabolism with high-flux glycolytic metabolism, generating alanine to support cellular growth, energy generation, and more energy-efficient nitrogen handling^20,35^. dFBA simulations predicted a two-phase process for nitrogen flow, the first phase being the release of abundant ammonia from oxidative deamination of amino acids during *Clostridial* Stickland fermentation (Figs. 3b,e,i and 4, reaction 18, nitrogen cycling)^25^. The second phase is characterized by the reassimilation of released ammonia by glutamate dehydrogenase and concomitant transamination of pyruvate to alanine (Figs. 3i and 4, reactions 17 and 18). HRMAS ^13^C NMR of *C. difficile* cultures grown with [U-^13^C]glucose and l-[^15^N]leucine confirmed model predictions of enriched nitrogen flow from leucine to alanine formed with carbon backbones from glycolytic pyruvate (Figs. 4 and 5c,d, nitrogen cycling). After the depletion of preferred Stickland acceptors (Fig. 3e, reactions 12 and 13), alanine’s use as a nitrogen sink consumes reducing equivalents, regenerating oxidized electron carriers for ATP-producing oxidative reactions in high-flux glycolytic and mixed-acid fermentations (Figs. 3i and 4, reactions 17 and 18)^35^ and further supports cellular systems in protein and peptidoglycan synthesis^36^, energy storage^28,37^ and osmotic balance^38,39^.

Live-cell HRMAS ^13^C NMR with dynamic metabolic modeling provides a unified methodology to define cellular-scale anaerobic metabolism for diverse applications. This analytic approach can support further analyses of prokaryotic physiology including microbial responses to antibiotics or optimization of conditions to produce bioactive or industrially important chemicals from different input feedstocks.

## Methods

### Strains

A pathogenicity locus (PaLoc)-deleted strain of *C. difficile* ATCC 43255 was generated that lacked the *tcdB*, *tcdE* and *tcdA* genes to reduce biohazard risks for NMR analyses. The deletion mutant was created using a toxin-mediated allele exchange method^40^. Briefly, approximately 800 bp of DNA flanking the pathogenicity locus were amplified by PCR from *C. difficile* ATCC 43255 using the primers in Supplementary Table 11. Purified PCR products were cloned into the PmeI site of the pMSR0 vector using NEBuilder HiFi DNA Assembly. The resulting plasmid was transformed into *E. coli* strain NEB10β (New England Biolabs) and insert verified by sequencing. *E. coli* strains were cultured aerobically at 37 °C in Luria-Bertani (LB) medium or LB agar supplemented with chloramphenicol (15 μg ml^−1^). The plasmid was then transformed into *E. coli* HB101 (RP4) and conjugated into *C. difficile* ATCC 43255 that had been heat-shocked at 50 °C for 15 min. Transconjugants were selected on Brain Heart Infusion (BHI) agar plates with cycloserine (250 μg ml^−1^), cefoxitin (25 μg ml^−1^) and thiamphenicol (15 μg ml^−1^). Allelic exchange was performed as described^40^. This strain was shown to be nontoxigenic to human fibroblasts using the methods described in Girinathan et al.^9^.

### Strain culture conditions

The ΔPaLoc strain of ATCC 43255 was cultured for 12 h in supplemented BHI media (Remel). Cells were spun and washed three times in prereduced PBS, prepared in molecular-clean water and diluted to introduce 100,000 cells into HRMAS NMR rotor inserts for analyses. Preparations were serially diluted and plated to *Brucella* agar (Remel) to quantitate vegetative cells and spores used in input preparations. Spore counts after 12 h of culture in supplemented BHI media were <0.1% of vegetative cells.

*C. difficile* MMM (pH 7.2) was prepared by supplementing *C. difficile* minimal medium (CDMM)^41^ with 100 µM sodium selenite and 0.5% glucose. MMM was prepared by substituting [U-^13^C]glucose (d-glucose-^13^C_6_, 99 atom), l-[U-^13^C]proline (l-proline-^13^C_5_, 99 atom %) or l-[U-^13^C]leucine (l-leucine-^13^C_6_, 98 atom %) in place of its natural carbon-isotope abundance analog at the same concentration (Sigma-Aldrich). Studies evaluating flow of amino nitrogen from Stickland-fermented leucine to [U-^13^C]pyruvate generated in glycolysis used l-[^15^N]leucine (Sigma-Aldrich).

HRMAS Kel-F rotor inserts (Bruker BioSpin Corporation) were loaded with approximately 100,000 CFU in an anerobic chamber to defined MMM. The insert was sealed and removed from the chamber for NMR analyses.

After analyses, rotor contents were checked for pH, serially diluted, plated to *Brucella* agar and incubated anaerobically at 37 °C for cellular biomass and absence of contaminating species. Contents were also Gram stained and visualized by optical microscopy to confirm cellular morphology. Cellular growth occurring in the rotors is shown in Supplementary Table 1. The pH remained ranged from 7.17 to 7.27 after 36 h of analyses.

### HRMAS NMR

HRMAS NMR measurements were performed on a Bruker Avance III HD 600 MHz spectrometer (Bruker BioSpin Corporation). The sealed Kel-F insert with live cells loaded in the anaerobic chamber was placed with 2 µl of D_2_O (for field locking) in a 4 mm zirconia rotor before the rotor was sealed and introduced into the triple-resonance HRMAS probe. One- and two-dimensional (1D, 2D) ^1^H and ^13^C NMR were conducted at 37 °C with a spin-rate of 3,600 ± 2 Hz. 1D time series spectra were measured alternately and continuously for ^1^H NOESY (NOE spectroscopy) with water suppression (roughly 13 min) and for proton-decoupled ^13^C (roughly 43 min) throughout the length of the experimental time. 2D ^1^H COSY (correlated spectroscopy, roughly 3 h and 49 min), proton-decoupled ^13^C COSY (roughly 3 h and 30 min) and ^13^C-decoupled ^1^H-^13^C HSQC (heteronuclear signal quantum coherence, roughly 3 h and 38 min) spectra were inserted in the between the 1D time series. Magnetic resonance spectra were processed using the TopSpin 3.6.2 (Bruker BioSpin Corporation), as well as with NUTS (Acorn NMR Inc.).

### Nonspinning control experiments

To evaluate the impact of spinning of the HRMAS NMR rotor on cellular morphology, metabolism and biomass, sealed HRMAS inserts were prepared as described in the section Strain culture conditions and incubated without spinning for the duration of runs that evaluated a paired insert subjected to HRMAS NMR. Single 1D and 1D ^1^H and ^13^C NMR spectra were acquired from the nonspinning insert after the end of the incubation period for comparison with the spun insert, as described in the section HRMAS NMR. Rotor contents were analyzed as described in the section Strain culture conditions (Supplementary Table 1). Analyses showed comparable metabolic profiles. By Gram stain, rotors maintained outside the NMR showed more advanced progression of cultures to a postsporulation state, as evidenced by the presence of spores and lysed mother cells (Extended Data Fig. 1).

### Identification of metabolite productions with 2D NMR

**Extended Data Fig. 6:**
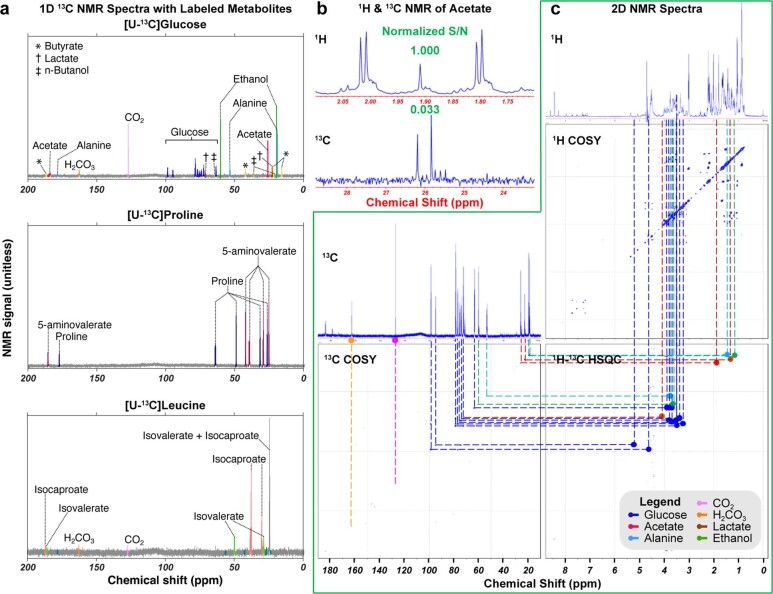
Live cell ^13^C NMR spectra and metabolite identification. (**a**) Proton-decoupled ^13^C NMR spectra of live cells following reactions with [U-^13^C]glucose, L-[U-^13^C]proline, and L-[U-^13^C]leucine, respectively. (**b**) ^1^H and proton-decoupled ^13^C NMR spectra of [U-^13^C]glucose produced ^13^C_2_-acetate, measured at 45.5 and 45.0 h, respectively. Relative signal-to-noise ratio (S/N) normalized to unit time for measured for ^1^H (S/N = 1.000) and ^13^C (S/N = 0.033). The double-doublet peaks seen in the ^1^H spectrum resulted from ^1^H J-couplings with both methyl and carboxyl ^13^C nuclei. The center peak represented methyl-^1^H bond to ^12^C. The doublet peaks seen in the ^13^C spectrum were the result of ^13^C-^13^C J-coupling between methyl and carboxyl ^13^C nuclei. (**c**) An illustration of metabolite identifications for live cells grown with [U-^13^C]glucose through 2D ^13^C COSY, ^13^C-^1^H HSQC, and ^1^H COSY according to reported ^13^C and ^1^H chemical shift values from the HMDB. In this illustration, the ^1^H 1D spectrum was measured at 45.5 h; ^1^H 2D COSY, at 32.6 h; ^1^H-^13^C HSQC, at 27.9 h; ^13^C COSY, at 37.2 h; and ^13^C 1D spectrum was a composite spectrum of all proton-decoupled ^13^C spectra (*N* = 11) measured between 24.8 and 45.0 h.

^13^C-labeled metabolites produced from [U-^13^C]glucose, l-[U-^13^C]proline and l-[U-^13^C]leucine in live cells were identified through 2D NMR (Extended Data Fig. 6) according to reported ^13^C and ^1^H chemical shift values availed from Human Metabolome Database (HMDB, https://hmdb.ca) and Biological Magnetic Resonance Bank (BMRB, https://bmrb.io).

### ^1^H and ^13^C spectra analyses

Individual free induction decay (.fid) files were processed using NMRPipe^42^, nmrglue^43^ and custom Python scripts available in the GitHub repository. Fourier transformed spectra were normalized by the noise root-mean-square error of the sparse 130–160 ppm region (Supplementary Figs. 2–4). The normalized spectrum stack was rendered as a surface plot in MATLAB R2019b (MathWorks) with face lightness mapped to the log_2_ of signal. Peaks with height ≥6 times the noise root-mean-square error (130–160 ppm) and separated from other peaks by 0.08 ppm were classified as detectable signals and assigned to compounds using the following algorithm:

1. Subpeaks within 0.3 ppm of each other were clustered.
2. Reference shifts for expected compounds within 0.45 ppm of the cluster were associated with the cluster.
3. All subpeaks from a cluster that associated with a single reference peak were assigned to the compound producing that reference peak.
4. In cases where a single cluster was associated with multiple reference peaks, subpeaks were manually assigned to compounds according to the reference chemical shifts from HMDB and associated splitting patterns. When the number of detected subpeaks was less than the expected multiplicity of the contributing reference peaks, extensive overlap was suspected and the clusters were excluded from analyses, as was the case for the isovalerate and isocaproate shifts at roughly 24.6 ppm in the [U-^13^C]leucine experiment. The remainder of manually assigned clusters used the 1D ^13^C and 1D ^1^H-^13^C HSQC spectra to deconvolute subpeaks in cases with resolvable overlap.

Assigned peak signals were concatenated into signal ridges, color-labeled by metabolite and superimposed over the stack as a scatter plot with stems to the *xy* plane and a smoothing spline curve fit. Surface regions within 0.5 ppm of each reference peak were colorized.

Assigned peaks in the ^13^C spectra ≤100 ppm were curve fit using a Voigt (Gaussian–Lorentzian convolution) lineshape and integrated. Logistic curve fits of metabolite integral versus time were calculated in Python using a least-squares regression (SciPy^44^ v.1.6.2) according to equation (1) (Supplementary Table 2). The peaks assigned to the acetate and ethanol methyl hydrogens in the ^1^H spectra from the [U-^13^C]glucose experiment were also curve fit to estimate the production of natural-abundance acetate.

### Selenium perturbation experiment

HRMAS NMR was performed as described above with cultures in MMM containing an excess of 30 mM l-[U-^13^C]proline, with or without 100 µM sodium selenite. Proline depletion was estimated as the ratio of final integrated [^13^C]proline signal to the starting integrated [^13^C]proline signal. Relative 5-aminovalerate production was estimated by comparting the ratios of final integrated [^13^C]5-aminovalerate signal to the starting integrated [U-^13^C]proline signal.

### Metabolic modeling

A previously published genome-scale metabolic model of *C. difficile* strain 630, icdf834 (ref. ^45^), was modified using the COBRApy toolbox^46^ and custom Python scripts. For metabolites not constrained by NMR trajectories, exchange reaction bounds were set to 3% of the millimolar concentration of media components used experimentally (Strain culture conditions). The cysteine uptake upper bound was set to 1,000 to enable uptake according to cellular demand. All other exchange reactions were blocked by setting their flux upper and lower bounds to zero.

icdf843, an updated model included in the GitHub repository, added the changes noted in Supplementary Table 4 (refs. ^5,9,20,28,35^), as supported by experimental data, and supported biologically relevant processes energetically and thermodynamically. Additions also included an exchange reaction for iron(II); transport and secretion reactions for propionate, phenylacetate, indole-3-acetate, butyrate, *n*-butanol and hydrogen sulfide; and proton-motive force dependent transport reactions for acetate, l-alanine, l-leucine, l-proline, l-isoleucine, isovalerate, isobutyrate, 2-methylbutyrate, isocaproate and 5-aminovalerate.

### Estimation of exchange fluxes for dFBA

While the direct relationship between relative proton abundance and peak area in the ^1^H-NMR spectrum can be leveraged to estimate the relative abundance of protons at specific molecular contexts in a sample, an equivalent relationship between ^13^C abundance and peak area does not exist in ^13^C NMR due to molecular context effects, including NOE cross-relaxation between adjacent protons and the ^13^C nuclei^21^. This property of ^13^C NMR prevents credible estimates of metabolite concentrations using relative signal intensities alone.

To overcome limitations in ^13^C NMR for estimating metabolite concentrations to constrain exchange fluxes, the following approaches were used. First, the ^13^C substrate’s logistic derivative equation (3) was scaled by its input concentration in MMM, resulting in an equation for estimated uptake flux of the substrate. We next evaluated the metabolites produced during a labeled substrate’s fermentation and their predicted origin(s) from single or multiple metabolic pathways. If pathway associations were unknown or incompletely defined, the more complex case of multiple pathway origins was used.

The simplest approach, used in cases where a 1:1 relationship between the labeled substrate and its product eliminates the need for a product flux constraint, estimates substrate exchange flux using a logistic equation scaled by a known input substrate concentration. In *C. difficile*, proline fermentation is well-defined and is only known to follow a single pathway, yielding 5-aminovalerate^4^, making it an ideal candidate for this approach. A small amount of proline is also used for protein synthesis. Given the known concentration of proline in MMM, we transformed the logistic derivative equation (2) for proline into a flux trajectory by multiplying it by the factor [Proline]_init_/(*L*_Proline_ + *C*_Proline_), where [Proline]_init_ is the initial proline concentration in the NMR run and *L*_Proline_ and *C*_Proline_ are logistic equation (1) coefficients for proline. We left the 5-aminovalerate flux unconstrained as Stickland fermenters carrying a proline reductase consume most proline through this metabolic pathway^4,25^, with relatively modest proline use for biomass production. This approach performs best for compounds such as proline that possess a single, well-defined fermentation pathway, removing the need for a constraint on the product’s exchange flux.

In cases where a labeled substrate is metabolized into multiple products, such as leucine’s fermentation via two separate pathways^5,6^, we estimated exchange fluxes for the products isocaproate and isovalerate by determining an expected ratio using GC with flame ionization detection. Volatile short-chain fatty acids were extracted and quantified from stationary phase cultures of *C. difficile* ATCC 43255 ΔPaLoc grown in MMM lacking isoleucine as described in Girinathan et al.^9^, as shown in Supplementary Table 12. Expected isocaproate and isovalerate yields (*Y*_Product_) per mole of leucine were estimated by taking the molar ratio of each product in the flame ionization detection readout to the input leucine. Flux trajectories for the NMR runs were then estimated by multiplying equation (2) for each product by the factor [Leucine]_init_ × *Y*_Product_/*L*_Product_, where [Leucine]_init_ is the initial leucine concentration in the NMR run.

**Extended Data Fig. 7:**
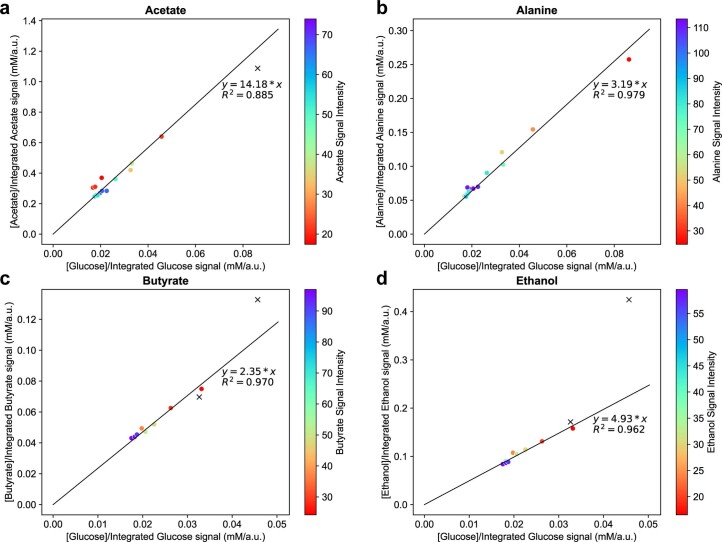
^13^C-signal enhancement standard curves of U-^13^C metabolites with respect to glucose. (**a**) Scatter plot of concentration-signal ratio of [U-^13^C]acetate versus [U-^13^C]glucose, determined from HRMAS ^13^C-NMR acquisitions of the solutions listed in Supplementary Table 9. Only peaks ≤ 100 ppm are included in the analyses; acquisitions where the average per-carbon peak area for a compound is less than 18 are marked with a black cross and excluded from the linear regression. Best fit line shown with equation and R-squared statistic. (**b**) Scatter plot of concentration-signal ratio of [U-^13^C]alanine versus [U-^13^C]glucose, determined as in panel (**a**). (**c**) Scatter plot of concentration-signal ratio of [U-^13^C]butyrate versus [U-^13^C]glucose, determined as in panel (**a**). (**d**) Scatter plot of concentration-signal ratio of [U-^13^C]ethanol versus [U-^13^C]glucose, determined as in panel (**a**).

In the third example, we estimated exchange fluxes of [U-^13^C]glucose metabolites using the input glucose concentration, the relative ^13^C NMR peak areas of glucose and each metabolite, and correction factors for NOE-induced signal enhancement empirically derived from standard solutions containing U-^13^C metabolites. Since glucose fermentation is highly integrated with central carbon metabolism, methods to estimate product concentration specific to the ^13^C input substrate are required, such as acetate originating from glucose versus glycine fermentation. For this case, we measured standard solutions within the dynamic range of our experiments under ^13^C NMR and integrated the peak areas to estimate a relationship between concentration and signal amplitude for each compound. As discussed in the Results section Dynamic FBA, ^13^C NMR signal amplitude is dependent on the molecular context of an individual ^13^C atom and is influenced by predictable factors, the most salient being ^1^H-^13^C NOE and spin rotation^21^. We assume that the relative effects of these properties on ^13^C NMR signal amplitude remain consistent between ^13^C atoms regardless of NMR acquisition parameters, and thus we define ^13^C NMR signal enhancement as the relative NMR signal-concentration ratio of each product with respect to [U-^13^C]glucose. Solutions listed in Supplementary Table 13 were measured by ^13^C NMR and processed using the procedures in HRMAS NMR and ^1^H and ^13^C spectra analyses above. Concentration-to-signal ratios of [U-^13^C]acetate, [U-^13^C]alanine, [U-^13^C]ethanol and [U-^13^C]butyrate (Sigma-Aldrich) in each standard solution were plotted versus the concentration-to-signal ratio of [U-^13^C]glucose in the same standard solution, then fit using a least-squares regression to the equation:

3 $$\begin{array}{*{20}{c}} {y = ax} \end{array}$$

where 1/*a* represents the ^13^C NMR signal enhancement of each metabolite with respect to glucose (Extended Data Fig. 7). When low-intensity peaks were excluded (peak area <18 normalized signal units per ppm), a strong correlation was observed for each compound (*R*^2^ values displayed on Extended Data Fig. 7), suggesting that the ^13^C NMR signal enhancement of each metabolite with respect to glucose is consistent across experiments, even under inconsistent acquisition parameters. A normalization scheme for each product was then derived as follows:

4 $$\begin{array}{*{20}{c}} {\left[ {{{{\mathrm{Product}}}}} \right]_{{\mathrm{est}}} = \left[ {{{{\mathrm{Glucose}}}}} \right] \times \frac{{S_{{{{\mathrm{Glucose}}}}}}}{{\left[ {{{{\mathrm{Glucose}}}}} \right]}} \times \frac{{S_{{{{\mathrm{Product}}}}}}}{{S_{{{{\mathrm{Glucose}}}}}}} \times \frac{{\left[ {{{{\mathrm{Product}}}}} \right]}}{{S_{{{{\mathrm{Product}}}}}}}} \end{array}$$

5 $$\begin{array}{*{20}{c}} {\left[ {{{{\mathrm{Product}}}}} \right]_{{\mathrm{est}}} = \left[ {{{{\mathrm{Glucose}}}}} \right] \times \frac{{S_{{{{\mathrm{Product}}}}}}}{{S_{{{{\mathrm{Glucose}}}}}}} \times \frac{{\frac{{\left[ {{{{\mathrm{Product}}}}} \right]}}{{S_{{\mathrm{Product}}}}}}}{{\frac{{\left[ {{{{\mathrm{Glucose}}}}} \right]}}{{S_{{{{\mathrm{Glucose}}}}}}}}}} \end{array}$$

6 $$\begin{array}{*{20}{c}} {\left[ {{{{\mathrm{Product}}}}} \right]_{{\mathrm{est}}} = \left[ {{{{\mathrm{Glucose}}}}} \right] \times \frac{{S_{{{{\mathrm{Product}}}}}}}{{S_{{{{\mathrm{Glucose}}}}}}} \times a} \end{array}$$

where [Product]_est_ is the estimated concentration of the product; [Glucose] is the known input concentration of [U-^13^C]glucose for the run; *S*_Product_/*S*_Glucose_ is the ratio of maximum integrated signal between the product and glucose, determined by *f*_Product_(36)/*f*_Glucose_(0) where *f* is the logistic equation (1) and *a* is the product-specific coefficient in equation (3). The logistic derivative equation (2) for each product was then multiplied by [Product]_est_/*L*_Product_ and the logistic derivative equation (2) for glucose was multiplied by [Glucose]/(*L*_Glucose_ + *C*_Glucose_) to transform the equations into flux trajectories. This approach is most suitable for compounds with multiple products where labeled products are commercially available or easily extracted.

In ^1^H-NMR, the multiplicity of protons can be estimated directly from the ratios of integrated signals. To estimate concentration and flux curves for natural-abundance acetate in the [U-^13^C]glucose run, the estimated concentration of ^13^C-acetate was multiplied by the ratio of integrated methyl proton signal between natural-abundance acetate and ^13^C-acetate.

The error in concentration and flux estimates was estimated by propagating the errors in the logistic coefficients as determined by the SciPy least-squares fit function. The function for standard error of the logistic equation (1) was defined as:

7 $$\begin{array}{*{20}{c}} {\delta f\left( x \right) = \sqrt {\left( {\frac{{\partial f}}{{\partial L}}\delta L} \right)^2 + \left( {\frac{{\partial f}}{{\partial k}}\delta k} \right)^2 + \left( {\frac{{\partial f}}{{\partial x_0}}\delta x_0} \right)^2 + \left( {\frac{{\partial f}}{{\partial C}}\delta C} \right)^2} } \end{array}$$

where:

8 $$\begin{array}{*{20}{c}} {\frac{{\partial f}}{{\partial L}} = \frac{{f - C}}{L}} \end{array}$$

9 $$\begin{array}{*{20}{c}} {\frac{{\partial f}}{{\partial k}} = \left(\, {f - C} \right)\left( {\frac{{\left( {x - x_0} \right){\mathrm{e}}^{-k\left( {x - x_0} \right)}}}{{1 + {\mathrm{e}}^{-k\left( {x - x_0} \right)}}}} \right)} \end{array}$$

10 $$\begin{array}{*{20}{c}} {\frac{{\partial f}}{{\partial x_0}} = \left(\, {f - C} \right)\left( {\frac{{-k{\mathrm{e}}^{-k\left( {x - x_0} \right)}}}{{1 + {\mathrm{e}}^{-k\left( {x - x_0} \right)}}}} \right)} \end{array}$$

11 $$\begin{array}{*{20}{c}} {\frac{{\partial f}}{{\partial C}} = 1} \end{array}$$

and 𝛿*L*, 𝛿*k*, 𝛿*x*_0_ and 𝛿*C* are the standard errors of the logistic coefficients. Likewise, the function for standard error of the logistic derivative equation (2) was defined as:

12 $$\begin{array}{*{20}{c}} {\delta f\,^{\prime} \left( x \right) = \sqrt {\left( {\frac{{\partial f^{\prime} }}{{\partial L}}\delta L} \right)^2 + \left( {\frac{{\partial f^{\prime} }}{{\partial k}}\delta k} \right)^2 + \left( {\frac{{\partial f^{\prime} }}{{\partial x_0}}\delta x_0} \right)^2} } \end{array}$$

where:

13 $$\begin{array}{*{20}{c}} {\frac{{\partial f^{\prime} }}{{\partial L}} = \frac{{f^{\prime} }}{L}} \end{array}$$

14 $$\begin{array}{*{20}{c}} {\frac{{\partial f^{\prime} }}{{\partial k}} = \left( f\,^{\prime} \right) \left( {\frac{1}{k} - x + x_0 + \frac{{2\left( {x - x_0} \right){\mathrm{e}}^{-k\left( {x - x_0} \right)}}}{{1 + {\mathrm{e}}^{-k\left( {x - x_0} \right)}}}} \right)} \end{array}$$

15 $$\begin{array}{*{20}{c}} {\frac{{\partial f^{\prime} }}{{\partial x_0}} = \left( f\,^{\prime} \right) \left( {\frac{{k{\mathrm{e}}^{-k\left( {x - x_0} \right)} - k}}{{1 + {\mathrm{e}}^{-k\left( {x - x_0} \right)}}}} \right)} \end{array}$$

### Aligning the time axes and calculating average logistic coefficients

The rate of isocaproate formation, as represented by the 0.864 ppm peak in the ^1^H-NMR time series, was selected as a normalization marker to align the time axes of all three runs because the 0.864 ppm isocaproate peak is well-separated in the ^1^H-NMR spectra, permitting reliable detection. A logistic curve (1) was fit to the 0.864 ppm isocaproate peak in the ^1^H time series of each run as described in the section ^1^H and ^13^C spectra analyses. The time axes for the three runs were normalized by aligning the time at which the logistic curve fit to the isocaproate ^1^H signal was 5% of its maximum value (Supplementary Figs. 1c,f,i, 2c,f,i and 3c,f,i).

Average logistic coefficients for all constrained metabolites were computed and averaged across three experimental replicates. Logistic coefficient errors were propagated as the root sum of squares (Supplementary Table 3). In cases where metabolites were not detected or signal was too low in a given run for successful logistic curve fitting, concentration estimates were scaled proportionally to the number of runs with signal supporting curve fitting.

### Dynamic FBA

dFBA was implemented by computing steady-state FBA and FVA solutions with time-dependent exchange fluxes using the COBRApy toolbox^46^ and custom Python scripts. Exchange (for input metabolites) and secretion (for end metabolites) upper and lower bounds were set to the logistic function derivatives (2) transformed as explained in the sections Estimation of exchange fluxes for dFBA and Aligning the time axes and calculating average logistic coefficients, evaluated per time point (Fig. 2b,d,f and Supplementary Tables 4 and 6). Leucine exchange flux was left unconstrained due to low model tolerances. Roughly 39% of glucose consumption was not accounted for in the formation of short-chain fatty acids and alcohols and was likely diverted to the production of biomass or exopolysaccharide, in agreement with previously reported results from Dannheim et al. The glucose exchange fluxes were therefore limited to allow only uptake of glucose at a rate expected to yield the observed short-chain products. Glucose shunted to anabolic processes was not included due to competition of these processes with the ATP hydrolysis objective.

Before simulation, additional constraints were placed on the model. Neumann-Schaal et al. demonstrate that in amino acid-rich media, 2-methylbutyrate secretion roughly coincides with 5-aminovalerate secretion and isobutyrate secretion roughly cooccurs with isocaproate secretion^23^. Thus, the secretion flux of 2-methylbutyrate was set to the isovalerate trajectory, shifted such that the half-maximum coefficient *x*_*0*_ equaled that of 5-aminovalerate, and scaled such that the upper asymptote *L* equaled the input isoleucine concentration, assuming complete fermentation. Likewise, the secretion flux of isobutyrate was locked to the isovalerate trajectory, shifted such that the half-maximum coefficient *x*_0_ equaled that of isocaproate and scaled by the GC-determined isobutyrate/isovalerate ratio (Supplementary Table 12). Additionally, acetyl-CoA synthase flux was locked to the secretion of butyrate, according to in vitro results and the metabolic mechanism proposed by Gencic et al.^35^. Last, the ^1^H-NMR derived curve for natural-abundance acetate flux was added to the ^13^C NMR curve for glucose-derived acetate, to account for acetate-forming reactions from nonglucose carbon sources, including the fermentations of cysteine and glycine and the Wood–Ljungdahl pathway.

ATP hydrolysis was selected for the biological objective as a common metabolic driver during log phase, stationary phase and sporulation (Supplementary Information). Steady-state FBA solutions were calculated along a simulated 36-h timescale with a resolution of five solutions per hour. FVA solutions were computed along the same simulated timescale with an objective flux threshold of 99.5%. Metabolic flux trajectories of selected reactions were visualized using custom MATLAB scripts (Fig. 3). Fluxes contributing to the production and consumption of cytosolic l-alanine, l-glutamate, ATP, pyruvate and ammonia were recorded at each timepoint (Supplementary Table 6, columns C-AL).

### Calculating flux proportions

Contributions to ATP influx were calculated by taking recorded reaction fluxes in Supplementary Table 6 as proportions of ATP flux as a whole:

1. The early-phase ATP-producing Oxidative Stickland flux was taken as the ratio of ATP-producing oxidative Stickland flux from 0 to 9.8 h (dark-green shading) to total ATP influx from 0 to 9.8 h (light- and dark-green shading).
2. The late-phase ATP-producing butyrate flux was taken as the ratio of butyrate kinase flux from 24 to 36 h (dark-orange shading) to total ATP influx from 24 to 36 h (light- and dark-orange shading).
3. The total glycolytic contribution to ATP production was calculated as the ratio of the red-boxed region to the total ATP influx from 0 to 36 h.
4. The glycolytic contribution to ATP production at each interval as the glycolytic flux in each interval (intersect of red box and each shaded region) divided by each shaded region’s flux (green, purple and orange for 0–9.8, 10–23.8 and 24–36 h, respectively).
5. The proportion of ATP production in each time interval was taken as the ratio of each shaded region’s flux (green, purple and orange for 0–9.8, 10–23.8 and 24–36 h, respectively) to total ATP influx.

Predicted amino nitrogen flow from l-leucine to l-alanine was estimated by the following scheme. We define two phases of ammonia metabolism: the deamination phase from 1 to 9 h where there is net secretion of ammonia, and the assimilation phase from 10 to 21 h where there is net uptake of ammonia. To accurately capture the l-alanine amino-group contribution from l-leucine at each timepoint, we considered amino groups transferred directly from leucine to alanine via glutamate, as well as leucine-origin ammonia released during the deamination phase and recycled onto alanine via glutamate during the assimilation phase.

We assume that the final step of leucine-alanine amino transfer is the transamination of glutamate and pyruvate by alanine transaminase. Therefore, at each timepoint, share of leucine-origin alanine is the proportion of leucine-origin nitrogen on glutamate multiplied by the total alanine transaminase flux. This share of leucine-origin glutamate consists of only glutamate produced by leucine transaminase during the deamination phase. During the assimilation phase, there is an additional component of glutamate synthesized by the reverse reaction of glutamate dehydrogenase from leucine-origin ammonia that was released during the deamination phase. This second component is estimated as the proportion of pooled ammonia that originated as leucine amino nitrogen multiplied by the glutamate dehydrogenase flux.

The total leucine-origin ammonia is estimated as the cumulative sum of leucine amino nitrogen that has been deaminated by the combined actions of leucine transaminase and the forward reaction of glutamate dehydrogenase. At each timepoint, this is the glutamate dehydrogenase flux multiplied by the leucine transaminase flux as a proportion of total glutamate influx. The proportion of pooled ammonia that originated as leucine amino nitrogen at the end of the deamination phase is estimated as the cumulative sum of leucine-origin ammonia divided by the total ammonia production during the deamination phase.

All of these calculations are recorded in Supplementary Table 6 under the leucine-alanine calculations heading.

### Analyses of ^15^N-^13^C peak splitting in ^13^C NMR spectral analyses of ^13^C alanine

For detection of ^15^N in the ^13^C spectrum, triplicate cultures of *C. difficile* ATCC 43255 *ΔPaLoc* were grown anaerobically in MMM containing [U-^13^C]glucose and either natural-abundance leucine or [^15^N]leucine. Cultures were centrifuged 48 h after inoculation and supernatants were collected, filter-sterilized, lyophilized and reconstituted in D_2_O. NMR data measured with Bruker BioSpin (Bruker Corporation) were analyzed with NUTS, 2D Pro (Acorn NMR Inc.). The .fid files were processed using 0.2 Hz line-broadening and were zero-filled before Fourier transformation. After the Fourier transformation, resonance peaks were quantified using curve fitting with Lorentzian line shapes. Curve fitting was first performed globally to determine the average peak width across all fitted peaks. The curve was then refitted using the width average and Lorentzian function (Extended Data Fig. 5).

### ^13^C NMR analyses of alanine’s α-carbon in media with [U-^13^C]glucose and [^15^N]leucine

**Extended Data Fig. 8:**
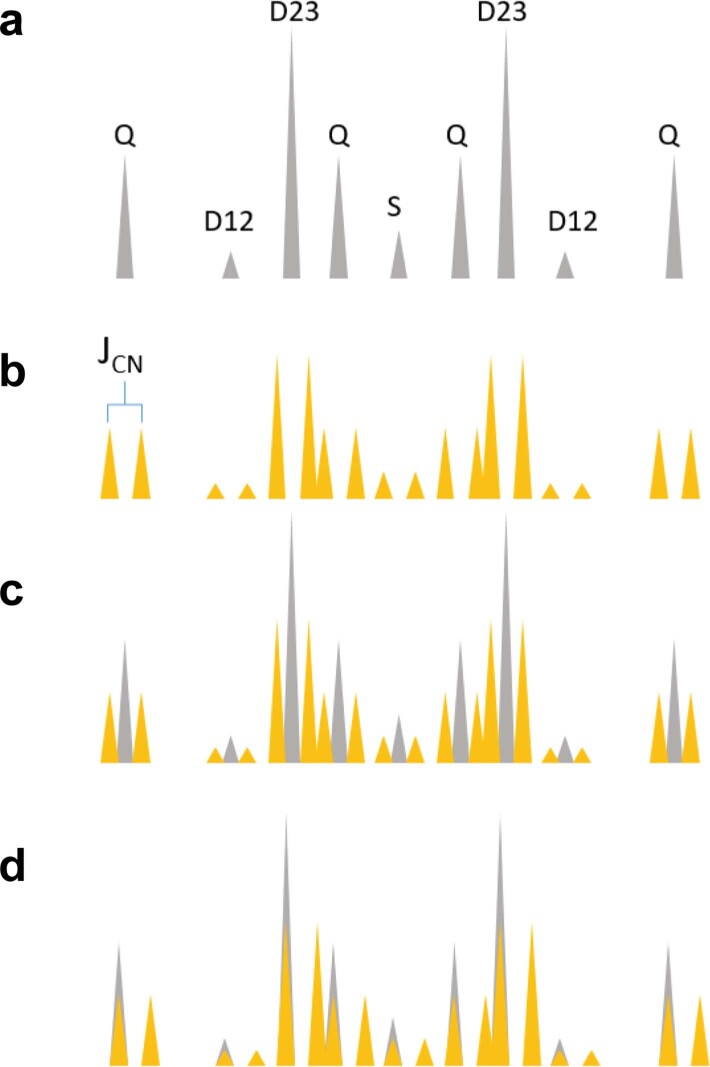
Deconvolution of alanine’s α-carbon in the ^13^C NMR spectral region. (**a**) All possible ^13^C patterns of alanine’s α-carbon, as produced from [U-^13^C]glucose, are shown in gray (Q: quadruple peaks from [U-^13^C]alanine, D12 and D23: doublets from [1,2-^13^C]alanine and [2,3-^13^C]alanine, respectively, S: singlet from [2-^13^C]alanine. (**b**) All peaks split into two equal intensity peaks due to ^15^N-^13^C J-coupling, shown in yellow. (**c**) The sum of peaks in A and B. (**d**) Predicted peak profile of all possible alanine species, with calculations that include shift of ^15^N associated ^13^C resonance due to isotope effects.

The complex peaks from alanine’s α-carbon in the ^13^C NMR spectral region can be deconvoluted based on (1) possible ^13^C label patterns (Extended Data Fig. 8a), where (a) ^13^C can appear on all three carbons in alanine that results in quadruple peaks occurring at the α-carbon; (b) carbon-13 can also appear on only two carbons in alanine: [1,2-^13^C]alanine and [2,3-^13^C]alanine, while [1,3-^13^C]alanine produces no detectable α-carbon-^13^C NMR signal and both two ^13^C-labeled isotopologs of alanine produce doublet peaks for the α-carbon and (c) carbon-13 only appears on the α-carbon as [2-^13^C]alanine to produce a singlet peak. (2) J-coupling of ^15^N-^13^C splits the α-carbon-^13^C NMR peak into two peaks when ^15^N is bound to α-^13^C (Extended Data Fig. 8b). (3) the isotope effect that introduces small shifts in the measured resonances^33^ (Extended Data Fig. 8d).

### Reporting summary

Further information on research design is available in the Nature Portfolio Reporting Summary linked to this article.

## Online content

Any methods, additional references, Nature Portfolio reporting summaries, source data, extended data, supplementary information, acknowledgements, peer review information; details of author contributions and competing interests; and statements of data and code availability are available at 10.1038/s41589-023-01275-9.

## Supplementary information

Supplementary Information Supplementary tables 1–3, 9 and 11–13 and figs. 1–4.

Reporting Summary

Supplementary Tables 4–8, 10 Supplementary Table 4: Modifications to icdf834, a previously published model of *C. difficile*^28^*.* The resulting model is named icdf843. Modifications are further elaborated in the Methods (Metabolic modeling). Supplementary Table 5: Evaluated exchange reaction bounds for NMR-measured metabolites. Uptake (proline, valine, isoleucine, and glucose) or secretion (all other metabolites) reaction upper and lower bounds as evaluated at each simulated timepoint, displayed in mM h^−1^. Supplementary Table 6: Selected dFBA-predicted metabolic fluxes. Predicted producing and consuming fluxes for l-alanine (alaL_c, producing: column C, consuming: column D), l-glutamate (gluL_c, producing: columns E-H, consuming: columns I-K), ATP (atp_c: columns L-S, consuming: columns T-Y), pyruvate (pyr_c: columns Z-AB, consuming: columns AC-AE), and ammonia (nh3_c: columns AF-AJ, consuming: columns AK-AL). The model objective flux is in column U. Calculations for estimated amino-group flux from leucine to alanine are in columns AM-BA. Supplementary Table 7: FVA simulation reaction lower bounds for all reactions in icdf843. Lower bounds for reactions accommodating an ATP hydrolysis objective flux within 0.5% of the optimal value. Flux units in mM h^−1^, table headers are reaction IDs. Supplementary Table 8: FVA simulation reaction upper bounds for all reactions in icdf843. Upper bounds for reactions accommodating an ATP hydrolysis objective flux within 0.5% of the optimal value. Flux units in mM h^−1^, table headers are reaction IDs. Supplementary Table 10: Pathways and enzyme systems involved in the metabolism of glucose, leucine, and proline in *C. difficile*. Column labels indicate the following information. Number, corresponding reaction numbers shown in Figs. 2 and 3. System or Enzyme, corresponding cellular system or enzyme-catalyzed pathway. Gene-association, CD630: associated geneID in the *C. difficile* reference strain CD630. Gene-association: ATCC43255, associated geneID in the *C. difficile* strain ATCC43255 used in the present studies.

## Data Availability

All NMR free induction decay files generated during the current study are available at the Metabolomics Workbench^47^ as study ST002433 (10.21228/M88M5G). The updated *C. difficile* metabolic model icdf843 is available on GitHub at https://github.com/Massachusetts-Host-Microbiome-Center/nmr-cdiff. The remaining data generated in this study are included in this article and its supplementary information files. Reference spectra used in this study to identify molecules in the NMR spectra were accessed from the HMDB (https://hmdb.ca/) and BMRB (https://bmrb.io) for l-proline (HMDB, HMDB0000162; BMRB, bmse000047), 5-aminovalerate (HMDB, HMDB0003355; BMRB, bmse000419), l-leucine (HMDB, HMDB0000687; BMRB, bmse000042), isovalerate (HMDB, HMDB0000718; BMRB, bmse000373), isocaproate (HMDB, HMDB0000689), d-glucose (HMDB, HMDB0000122; BMRB, bmse000015), acetate (HMDB, HMDB0000042; BMRB, bmse000191), ethanol (HMDB, HMDB0000108; BMRB, bmse000297), l-alanine (HMDB, HMDB0000161; BMRB, bmse000994), l-lactate (HMDB, HMDB0000190; BMRB, bmse000269), butyrate (HMDB, HMDB0000039; BMRB, bmse000402) and *n*-butanol (HMDB, HMDB0004327; BMRB, bmse000447).

## Code availability

All custom code developed for this study is available on GitHub at https://github.com/Massachusetts-Host-Microbiome-Center/nmr-cdiff.
