# Supplementary material for: Elucidating dynamic anaerobe metabolism with HRMAS 13C NMR and genome-scale modeling
Source: Nat Chem Biol. 2023 Mar 9;19(5):556–64. doi: 10.1038/s41589-023-01275-9 (PMC10154198; doi:10.1038/s41589-023-01275-9)
Supplement: Supplementary file 1 — Supplementary tables 1–3, 9 and 11–13 and figs. 1–4. [file 41589_2023_1275_MOESM1_ESM.pdf]

nature chemical biology

Article
https://doi.org/10.1038/s41589-023-01275-9

Elucidating dynamic anaerobe metabolism with HRMAS ^{13}C NMR and genome-scale modeling

In the format provided by the authors and unedited

This file contains:

Supplementary Table 1: *C. difficile* biomass after incubation in MMM in stationary or HRMAS acquisition conditions.

Supplementary Table 2: Logistic fit coefficients with standard error for integrated ^{13}C-NMR signal vs. time.

Supplementary Table 3: Logistic coefficients for the estimated concentration curves constraining dFBA analyses.

Supplementary Table 9. Isotopologue proportions of alanine in stationary phase *C. difficile* cultures grown in media containing [U-^{13}C]glucose and [^{15}N]leucine.

Supplementary Table 11: Primers used for construction of the PaLoc- strain.

Supplementary Table 12: Concentrations of isovalerate, isocaproate, and isobutyrate determined by gas chromatography with flame ionization detection (GC-FID).

Supplementary Table 13: Composition of standard solutions containing [U-^{13}C]glucose and selected metabolites at defined concentrations.

Supplementary Figure 1: Processed NMR datasets of *C. difficile* cultures grown in MMM with L-[U-^{13}C]Proline.

Supplementary Figure 2: Processed NMR datasets of *C. difficile* cultures grown in MMM with L-[U-^{13}C]Leucine.

Supplementary Figure 3: Processed NMR datasets of *C. difficile* cultures grown in MMM with [U-^{13}C]Glucose.

Supplementary Figure 4: dFBA predicted reaction fluxes for selected reactions across simulations with three objective functions.

Supplementary Table 1: *C. difficile* biomass after incubation in MMM in stationary or HRMAS acquisition conditions for the duration specified. Biomass represents the mean of three technical replicates of rotor contents incubated anaerobically on Brucella agar at 37°C for 48 hours. Uncertainty denotes the bounds of the 95% confidence interval of the mean. *Below Limit of Detection (BLD) denotes no viable biomass detected; the upper bound on biomass given is half the expected viable biomass yielding one colony on an agar plate at the lowest dilution factor.

Experiment	Incubation duration (h)	Condition	Biomass (CFU/mL)
1	36	Stationary	$(1.6 \pm 0.1) \times 10^5$
		HRMAS	$(6.7 \pm 1.3) \times 10^5$
2	36	Stationary	$(2.5 \pm 0.3) \times 10^5$
		HRMAS	$(8.2 \pm 0.4) \times 10^6$
3	76	Stationary	BLD (< 500)*
		HRMAS	BLD (< 500)*

Supplementary Table 2: Logistic fit coefficients with standard error. All curves are fit to trajectories of integrated ^{13}C-NMR signal vs. time, except for glucose-origin acetate (^{13}C-Acetate (C2)) and natural abundance non-glucose acetate (NA-Acetate (C2)), which were fit to trajectories of integrated ^{1}H-NMR methyl proton signal vs. time. *Coefficients excluded where logistic curves were not computed due to insufficient signal.

	**Replicate 1**				**Replicate 2**				**Replicate 3**			
**Metabolite**	***L***	***k***	***x0***	***C***	***L***	***k***	***x0***	***C***	***L***	***k***	***x0***	***C***
**Proline**	165.537 ± 2.101	-1.957 ± 0.095	6.296 ± 0.030	-0.190 ± 0.420	195.364 ± 1.639	-1.628 ± 0.083	7.119 ± 0.037	-0.296 ± 0.655	207.665 ± 1.816	-1.526 ± 0.065	5.140 ± 0.033	0.369 ± 0.557
5-amino-valerate	178.691 ± 0.512	2.077 ± 0.132	6.444 ± 0.038	—	223.807 ± 0.648	1.787 ± 0.080	7.188 ± 0.030	—	246.580 ± 0.830	1.694 ± 0.084	5.114 ± 0.034	—
**Leucine**	267.553 ± 6.030	-1.019 ± 0.095	6.643 ± 0.106	-1.829 ± 2.439	281.874 ± 3.218	-0.825 ± 0.039	7.718 ± 0.067	-0.902 ± 1.151	266.383 ± 2.309	-1.208 ± 0.053	7.422 ± 0.042	-0.443 ± 0.876
Isovalerate	14.219 ± 0.423	1.932 ± 0.766	4.590 ± 0.241	—	—*				—*			
Isocaproate	75.773 ± 0.634	1.963 ± 0.208	7.699 ± 0.062	—	90.066 ± 0.573	1.174 ± 0.096	8.562 ± 0.079	—	87.951 ± 0.649	1.145 ± 0.114	8.203 ± 0.099	—
**Glucose**	1407.38 ± 62.666	-0.187 ± 0.016	16.163 ± 0.415	191.084 ± 29.225	1539.90 ± 66.897	-0.185 ± 0.012	14.160 ± 0.416	418.705 ± 17.206	1477.95 ± 100.983	-0.179 ± 0.017	11.763 ± 0.713	826.109 ± 22.337
Acetate	773.609 ± 11.678	0.211 ± 0.019	16.240 ± 0.496	—	779.190 ± 11.765	0.243 ± 0.022	13.799 ± 0.399	—	498.538 ± 4.155	0.511 ± 0.052	9.982 ± 0.227	—
Alanine	334.628 ± 6.416	0.309 ± 0.046	14.464 ± 0.555	—	501.037 ± 3.229	0.351 ± 0.016	15.432 ± 0.145	—	398.810 ± 2.610	0.341 ± 0.021	14.049 ± 0.207	—
Ethanol	797.009 ± 10.735	0.190 ± 0.013	22.008 ± 0.422	—	444.417 ± 5.798	0.215 ± 0.012	22.336 ± 0.302	—	338.553 ± 3.719	0.197 ± 0.014	21.843 ± 0.404	—
Butyrate	136.042 ± 3.073	0.258 ± 0.033	28.571 ± 0.573	—	—*				—*			
**^{13}C-Acetate (C2)**	680.230 ± 22.342	0.271 ± 0.033	15.563 ± 0.548	—	465.351 ± 6.896	0.351 ± 0.029	11.109 ± 0.268	—	714.158 ± 11.237	0.273 ± 0.017	14.964 ± 0.272	714.158 ± 11.237
NA-Acetate (C2)	167.411 ± 4.848	0.441 ± 0.153	3.570 ± 0.806	—	54.910 ± 1.085	1.110 ± 0.407	7.488 ± 0.376	—	—*			

Supplementary Table 3: Logistic coefficients for the estimated concentration curves constraining dFBA analyses. *Leucine exchange was bounded by [0, 1000] mM/h at all timepoints. †Glucose flux was bounded by the butyrate curve plus half the sum of acetate, alanine, ethanol curves. ‡Acetate flux was bounded by the sum of the glucose-origin acetate and non-glucose natural abundance (NA) acetate curves.

**Metabolite**	***L***	***k***	***x0***	***C***
**Proline**	6.962 ± 0.122	-1.704 ± 0.141	6.185 ± 0.058	-0.002 ± 0.035
5-aminovalerate	6.960 ± 0.037	1.853 ± 0.176	6.249 ± 0.059	—
**Leucine***	N/A			
Isovalerate	0.933 ± 0.056	1.519 ± 0.366	4.533 ± 0.182	—
Isocaproate	4.830 ± 0.052	1.375 ± 0.178	8.106 ± 0.131	—
Valine	1.297 ± 0.026	-1.519 ± 0.366	8.106 ± 0.131	—
Isobutyrate	1.297 ± 0.026	1.519 ± 0.366	8.106 ± 0.131	—
Isoleucine	2.289 ± 0.046	-1.519 ± 0.366	6.249 ± 0.059	—
2-methylbutyrate	2.289 ± 0.046	1.519 ± 0.366	6.249 ± 0.059	—
**Glucose†**	N/A			
Acetate‡	10.235 ± 0.251	0.323 ± 0.059	13.180 ± 0.631	—
Alanine	5.888 ± 0.127	0.340 ± 0.059	14.590 ± 0.614	—
Ethanol	8.064 ± 0.213	0.201 ± 0.023	22.108 ± 0.665	—
Butyrate	0.810 ± 0.052	0.271 ± 0.034	28.125 ± 0.538	—
Acetate (NA) ‡	1.632 ± 0.101	0.775 ± 0.435	5.529 ± 0.889	—

Supplementary Table 9. Isotopologue proportions of alanine in stationary phase *C. difficile* cultures grown in media containing [U-^{13}C]glucose and [^{15}N]leucine.

Alanine	Run1	Run2	Run3	AVG	STDEV	SEM
**U-^{13}C, ^{14}N**	19%	24%	23%	22%	2.65%	1.53%
**2,3-^{13}C, ^{14}N**	17%	22%	24%	21%	3.61%	2.08%
**U-^{13}C, ^{15}N**	36%	27%	27%	30%	5.20%	3.00%
**2,3-^{13}C, ^{15}N**	28%	26%	26%	27%	1.15%	0.67%
**TOTAL ^{14}N**	36%	46%	47%	43%	6.08%	3.51%
**TOTAL ^{15}N**	64%	53%	53%	57%	6.35%	3.67%

Supplementary Table 11: Primers used for construction of the PaLoc- strain.

Primer	Sequence (5' to 3')*	Use
BD013	ttttttgttaccctaagtttGGATGATTTTATGCAAAAGTC	5' left arm for *tcdBEA* deletion
BD014	tattttagccCATAAAATTTTCTCCTTTACTATAATATTTTTAC	3' left arm for *tcdBEA* deletion
BD015	aaattttatgGGCTAAAATATATGTTTGATAAAAAATTATTC	5' right arm for *tcdBEA* deletion
BD016	agattatcaaaaaggagtttCCAGCTTGTTCTGAAGAC	3' right arm for *tcdBEA* deletion
BD017	GGAGGATATATAAAAGAGTTTTAGC	5' screening of *tcdBEA* deletion
BD018	GGGTATTGCTCTACTGGC	3' screening of *tcdBEA* deletion

*Lowercase bases indicate overlapping sequences

Supplementary Table 12: Concentrations of isovalerate, isocaproate, and isobutyrate determined by gas chromatography with flame ionization detection (GC-FID).

	Volatile acids			Proportions			
**Condition**	**Isovalerate (iVal)**	**Isocaproate (iCap)**	**Isobutyrate (iBut)**	$\frac{iCap}{iVal}$	$\frac{iCap}{iCap + iVal}$	$\frac{iVal}{iCap + iVal}$	$\frac{iBut}{iVal}$
A	2.956	5.069	1.383	1.715	0.632	0.368	0.468
B	2.899	4.899	1.361	1.690	0.628	0.372	0.469
C	2.973	5.218	1.357	1.755	0.637	0.363	0.456
D	3.146	5.485	1.447	1.743	0.635	0.365	0.460
**Average**	**2.994**	**5.168**	**1.387**	**1.726**	**0.633**	**0.367**	**0.463**

Supplementary Table 13: Composition of standard solutions containing [U-^{13}C]glucose and selected metabolites at defined concentrations.

Solution	Concentration (mM)				
	Glucose	Ethanol	Acetate	Alanine	Butyrate
**A**	26.85	0.00	0.00	0.00	0.00
**B**	19.35	0.00	6.43	6.52	0.00
**C**	11.85	2.39	14.07	12.72	2.72
**D**	5.87	5.22	17.96	14.78	5.45
**E**	2.72	6.85	18.85	15.22	7.49
**F**	10.00	10.00	13.77	10.00	12.52

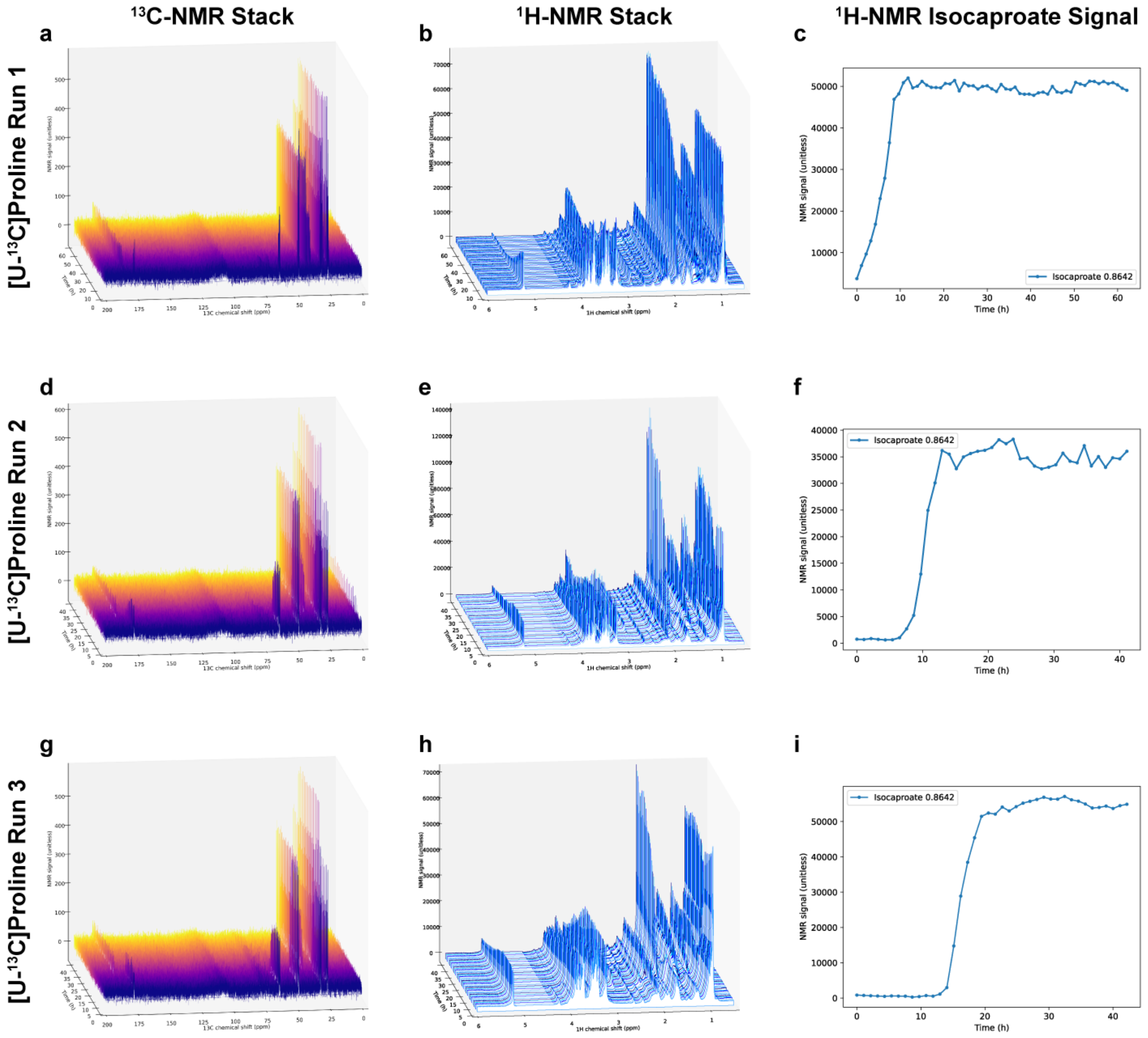

Supplementary Figure 1: Processed NMR datasets of *C. difficile* cultures grown in MMM with L-[U-^{13}C]Proline. (**a**) Waterfall plot of ^{13}C-NMR time series for experimental replicate 1, depicting unitless NMR signal (Z-axis) vs. chemical shift in ppm (X-axis) vs. time in hours (Y-axis). (**b**) Waterfall plot of ^{1}H-NMR time series for experimental replicate 1. Water resonances in spectra (4.35–5.15 ppm) were set to zero. Axes as in (a). (**c**) Line plot of isocaproate methyl proton signal for experimental replicate 1, depicting unitless NMR signal at 0.8642 ppm (Y-axis) vs. time in hours (X-axis). (**d**) Waterfall plot of ^{13}C-NMR time series for experimental replicate 2, axes as in (a). (**e**) Waterfall plot of ^{1}H-NMR time series for experimental replicate 2, processing and axes as in (b). (**f**) Line plot of isocaproate methyl proton signal for experimental replicate 2, axes as in (c). (**g**) Waterfall plot of ^{13}C-NMR time series for experimental replicate 3, axes as in (a). (**h**) Waterfall plot of ^{1}H-NMR time series for experimental replicate 3, processing and axes as in (b). (**i**) Line plot of isocaproate methyl proton signal for experimental replicate 3, axes as in (c).

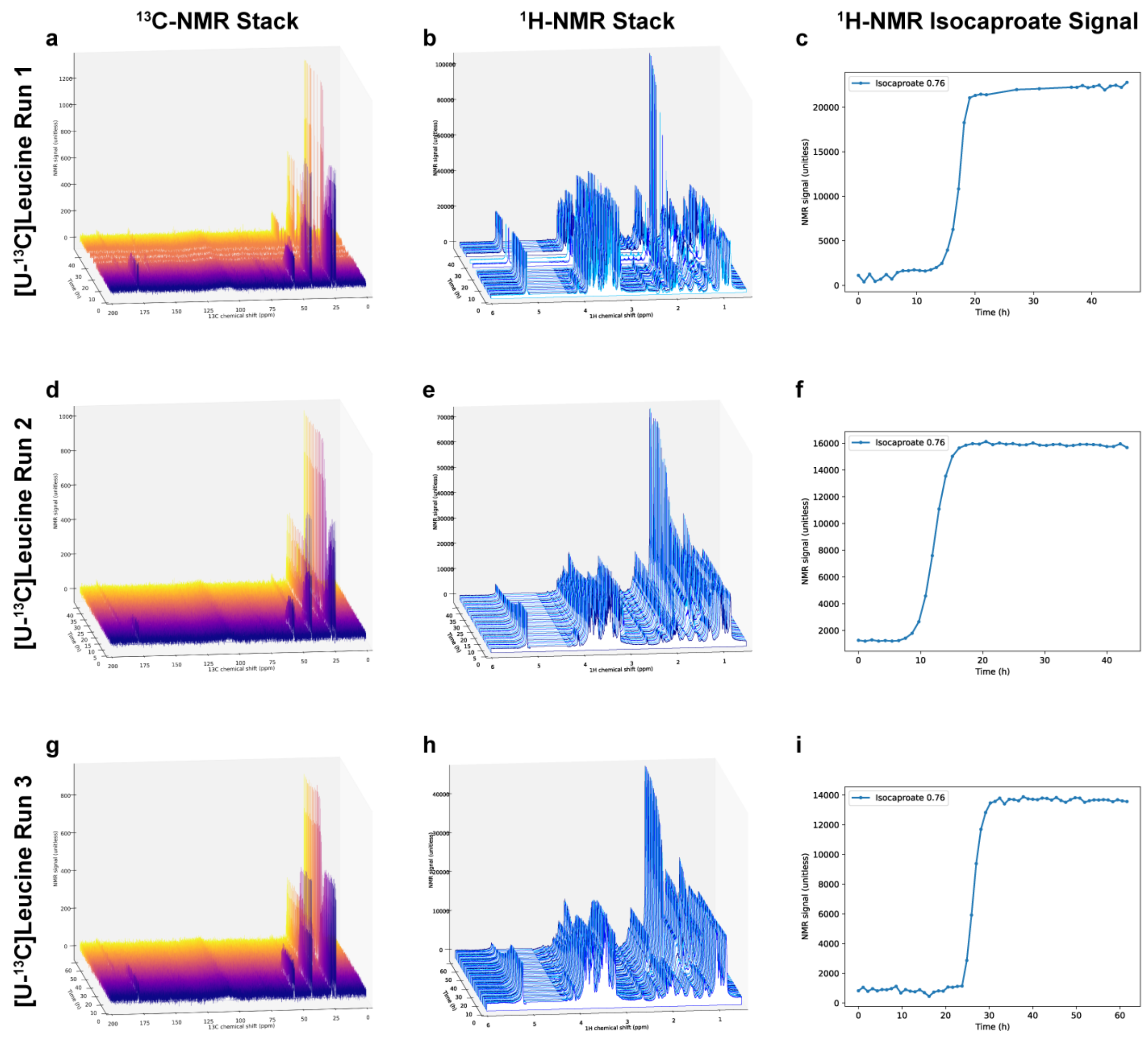

Supplementary Figure 2: Processed NMR datasets of *C. difficile* cultures grown in MMM with L-[U-^{13}C]Leucine. (**a**) Waterfall plot of ^{13}C-NMR time series for experimental replicate 1, depicting unitless NMR signal (Z-axis) vs. chemical shift in ppm (X-axis) vs. time in hours (Y-axis). (**b**) Waterfall plot of ^{1}H-NMR time series for experimental replicate 1. Water resonances in spectra (4.35–5.15 ppm) were set to zero. Axes as in (a). (**c**) Line plot of isocaproate methyl proton signal for experimental replicate 1, depicting unitless NMR signal at 0.760 ppm (Y-axis) vs. time in hours (X-axis). Note: in the [U-^{13}C]Leucine experiments, ^{1}H-^{13}C J-coupling effects split the isocaproate methyl proton signal into two subpeaks at 0.760 and 0.968 ppm. We selected the 0.760 ppm subpeak for measurement per its discrete separation from other spectral peaks. (**d**) Waterfall plot of ^{13}C-NMR time series for experimental replicate 2, axes as in (a). (**e**) Waterfall plot of ^{1}H-NMR time series for experimental replicate 2, processing and axes as in (b). (**f**) Line plot of isocaproate methyl proton signal for experimental replicate 2, axes as in (c). (**g**) Waterfall plot of ^{13}C-NMR time series for experimental replicate 3, axes as in (a). (**h**) Waterfall plot of ^{1}H-NMR time series for experimental replicate 3, processing and axes as in (b). (**i**) Line plot of isocaproate methyl proton signal for experimental replicate 3, axes as in (c).

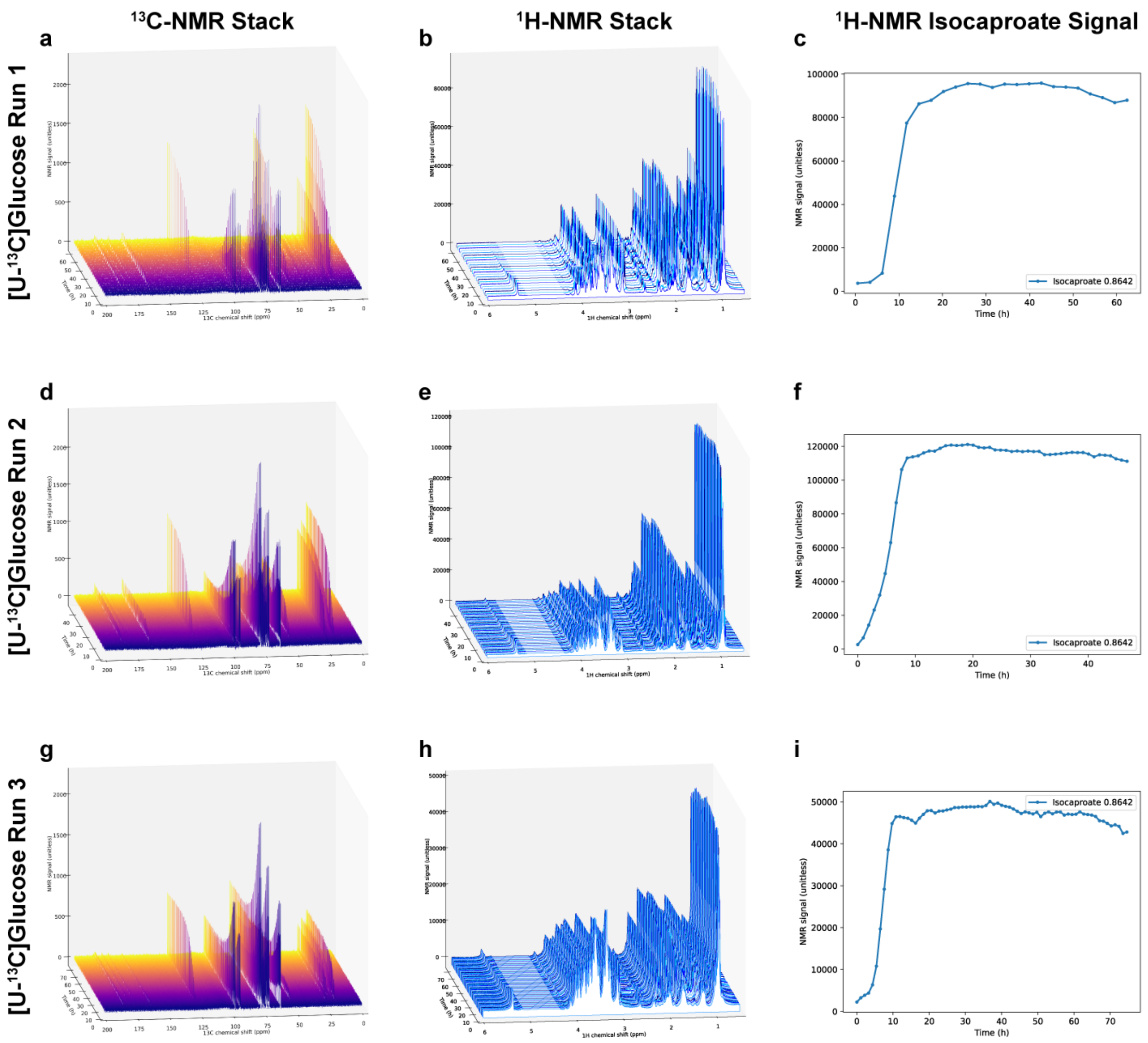

Supplementary Figure 3: Processed NMR datasets of *C. difficile* cultures grown in MMM with [U-^{13}C]Glucose. (**a**) Waterfall plot of ^{13}C-NMR time series for experimental replicate 1, depicting unitless NMR signal (Z-axis) vs. chemical shift in ppm (X-axis) vs. time in hours (Y-axis). (**b**) Waterfall plot of ^{1}H-NMR time series for experimental replicate 1. Water resonances in spectra (4.35–5.15 ppm) were set to zero. Axes as in (a). (**c**) Line plot of isocaproate methyl proton signal for experimental replicate 1, depicting unitless NMR signal at 0.8642 ppm (Y-axis) vs. time in hours (X-axis). (**d**) Waterfall plot of ^{13}C-NMR time series for experimental replicate 2, axes as in (a). (**e**) Waterfall plot of ^{1}H-NMR time series for experimental replicate 2, processing and axes as in (b). (**f**) Line plot of isocaproate methyl proton signal for experimental replicate 2, axes as in (c). (**g**) Waterfall plot of ^{13}C-NMR time series for experimental replicate 3, axes as in (a). (**h**) Waterfall plot of ^{1}H-NMR time series for experimental replicate 3, processing and axes as in (b). (**i**) Line plot of isocaproate methyl proton signal for experimental replicate 3, axes as in (c).

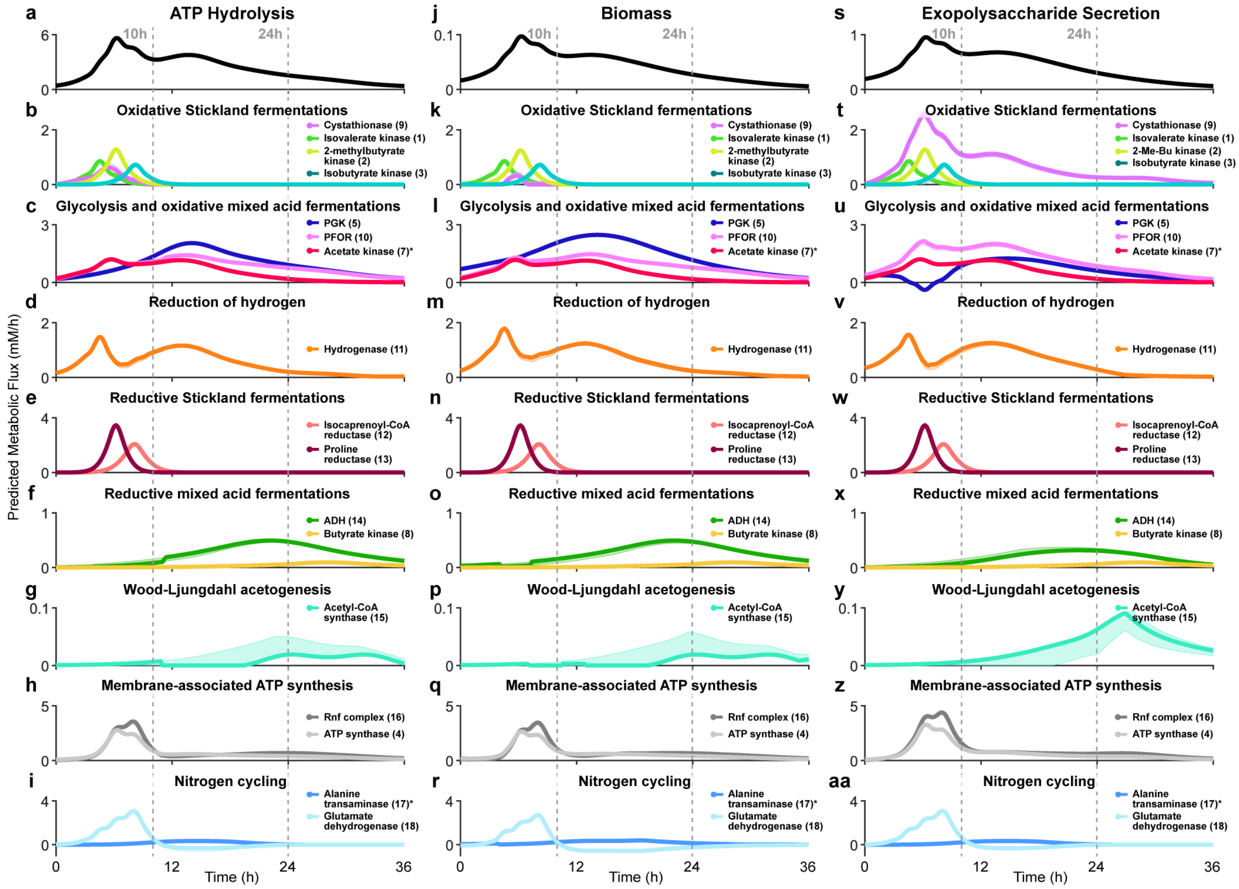

Supplementary Figure 4: dFBA predicted reaction fluxes for selected reactions across simulations with three objective functions. (**a-i**) dFBA predicted reactions fluxes over 36h of metabolism (X-axis) with ATP hydrolysis as the objective function. Y-axis shows inferred flux in mM/h. (**a**) ATP Hydrolysis flux. (**b-i**) Selected categorized reaction fluxes as in Fig. 3b-i. (**j-r**) dFBA predicted reactions fluxes over 36h of metabolism with biomass as the objective function. Axes as in (a-i). (**j**) Biomass flux. (**k-r**) Selected categorized reaction fluxes as in Fig. 3b-i. (**s-aa**) dFBA predicted reactions fluxes over 36h of metabolism with exopolysaccharide secretion as the objective function. Axes as in (a-i). (**s**) Exopolysaccharide secretion flux. (**t-aa**) Selected categorized reaction fluxes as in Fig. 3b-i.
