# Supplementary material for: Elucidating dynamic anaerobe metabolism with HRMAS 13C NMR and genome-scale modeling
Source: Nat Chem Biol. 2023 Mar 9;19(5):556–64. doi: 10.1038/s41589-023-01275-9 (PMC10154198; doi:10.1038/s41589-023-01275-9)
Supplement: Supplementary file 2 — Reporting Summary [file 41589_2023_1275_MOESM2_ESM.pdf]

nature portfolio

Corresponding author(s): Lynn Bry

Last updated by author(s): Jan 7, 2023

Reporting Summary

Nature Portfolio wishes to improve the reproducibility of the work that we publish. This form provides structure for consistency and transparency in reporting. For further information on Nature Portfolio policies, see our Editorial Policies and the Editorial Policy Checklist.

Statistics

For all statistical analyses, confirm that the following items are present in the figure legend, table legend, main text, or Methods section.

n/a	Confirmed	
☐	☒	The exact sample size (*n*) for each experimental group/condition, given as a discrete number and unit of measurement
☐	☒	A statement on whether measurements were taken from distinct samples or whether the same sample was measured repeatedly
☐	☒	The statistical test(s) used AND whether they are one- or two-sided<br>*Only common tests should be described solely by name; describe more complex techniques in the Methods section.*
☐	☒	A description of all covariates tested
☒	☐	A description of any assumptions or corrections, such as tests of normality and adjustment for multiple comparisons
☐	☒	A full description of the statistical parameters including central tendency (e.g. means) or other basic estimates (e.g. regression coefficient) AND variation (e.g. standard deviation) or associated estimates of uncertainty (e.g. confidence intervals)
☒	☐	For null hypothesis testing, the test statistic (e.g. *F*, *t*, *r*) with confidence intervals, effect sizes, degrees of freedom and *P* value noted<br>*Give P values as exact values whenever suitable.*
☒	☐	For Bayesian analysis, information on the choice of priors and Markov chain Monte Carlo settings
☒	☐	For hierarchical and complex designs, identification of the appropriate level for tests and full reporting of outcomes
☒	☐	Estimates of effect sizes (e.g. Cohen's *d*, Pearson's *r*), indicating how they were calculated

Our web collection on statistics for biologists contains articles on many of the points above.

Software and code

Policy information about availability of computer code

Data collection	MRS spectra were collected using TopSpin 3.6.2.
Data analysis	MRS spectra were processed using TopSpin 3.6.2 and NUTS (update 2/2/17). Data analysis was performed in MATLAB R2019b and Python 3.9.7 using custom scripts available on GitHub at https://github.com/Massachusetts-Host-Microbiome-Center/nmr-cdiff. Software dependencies are NMRPipe (Version 10.9 Revision 2021.258.11.26) and the Python packages nmrglue (0.9.dev0), SciPy (1.6.2), COBRApy (0.25.0), and other Python packages not listed in the manuscript but included requirements.txt in the GitHub repository, to facilitate the construction of a virtual environment containing all Python dependencies. Custom scripts provided in the GitHub repository accomplish the semi-automated processing of NMR spectra using NMRPipe and nmrglue, the estimation of concentrations from NMR signal trajectories, and the execution of dynamic Flux Balance Analysis simulations using COBRApy. The procedures for analysis are described extensively in the methods and in the GitHub repository documentation.

For manuscripts utilizing custom algorithms or software that are central to the research but not yet described in published literature, software must be made available to editors and reviewers. We strongly encourage code deposition in a community repository (e.g. GitHub). See the Nature Portfolio guidelines for submitting code & software for further information.

Data

Policy information about availability of data

All manuscripts must include a data availability statement. This statement should provide the following information, where applicable:

- Accession codes, unique identifiers, or web links for publicly available datasets
- A description of any restrictions on data availability
- For clinical datasets or third party data, please ensure that the statement adheres to our policy

All NMR free induction decay files generated during the current study are available at the Metabolomics Workbench47 as study ST002433 (http://dx.doi.org/10.21228/M88M5G). The updated C. difficile metabolic model icdf843 is available on GitHub at https://github.com/Massachusetts-Host-Microbiome-Center/nmr-cdiff. The remaining data generated in this study are included in this article and its supplementary information files.

Reference spectra used in this study to identify molecules in the NMR spectra were accessed from HMDB (https://hmdb.ca/) and BMRB (https://bmrb.io) for L-proline (HMDB: HMDB0000162; BMRB: bmse000047), 5-aminovalerate (HMDB: HMDB0003355; BMRB: bmse000419), L-leucine (HMDB: HMDB0000687; BMRB: bmse000042), isovalerate (HMDB: HMDB0000718; BMRB: bmse000373), isocaproate (HMDB: HMDB0000689), D-glucose (HMDB: HMDB0000122; BMRB: bmse000015), acetate (HMDB: HMDB0000042; BMRB: bmse000191), ethanol (HMDB: HMDB0000108; BMRB: bmse000297), L-alanine (HMDB: HMDB0000161; BMRB: bmse000994), L-lactate (HMDB: HMDB0000190; BMRB: bmse000269), butyrate (HMDB: HMDB0000039; BMRB: bmse000402), and n-butanol (HMDB: HMDB0004327; BMRB: bmse000447).

Human research participants

Policy information about studies involving human research participants and Sex and Gender in Research.

Reporting on sex and gender	n/a
Population characteristics	n/a
Recruitment	n/a
Ethics oversight	n/a

Note that full information on the approval of the study protocol must also be provided in the manuscript.

Field-specific reporting

Please select the one below that is the best fit for your research. If you are not sure, read the appropriate sections before making your selection.

☒ Life sciences ☐ Behavioural & social sciences ☐ Ecological, evolutionary & environmental sciences

For a reference copy of the document with all sections, see nature.com/documents/nr-reporting-summary-flat.pdf

Life sciences study design

All studies must disclose on these points even when the disclosure is negative.

Sample size	Three independent biological replicates were performed for all NMR studies supporting dFBA analyses or measuring 14N/15N isotopic composition of alanine. Measurement of three replicates enables assessment of variability and confirmation of consistency between repeat experiments, and is a widely accepted standard for experiments measuring bacterial growth in vitro.
Data exclusions	No data was excluded from this study.
Replication	Three biological replicates were performed for NMR time-series studies supporting dFBA analyses. Biological replicates indicated consistent metabolic profiles in repeated experiments, with the exception of butyrate only being detected in one and isovalerate only being detected in two replicates. We accounted for this by scaling down the estimated flux curves for those compounds in our dFBA analyses.<br><br>Three biological replicates were prepared for the experiments measuring 14N/15N isotopic composition of alanine as described in the section "Confirmation of metabolic integration between glycolytic and Stickland metabolism". The three biological replicates measured the 15N proportion with a standard deviation of 4%, demonstrating high biological reproducibility of the results.
Randomization	Randomization was not applicable because the study did not involve assignment of individuals to treatment groups. All microbiological studies used the C. difficile ATCC 43255 del-PaLoc strain from homogeneous spore stocks.
Blinding	Blinding was not applicable because the study did not involve assignment of individuals to treatment groups.

Reporting for specific materials, systems and methods

We require information from authors about some types of materials, experimental systems and methods used in many studies. Here, indicate whether each material, system or method listed is relevant to your study. If you are not sure if a list item applies to your research, read the appropriate section before selecting a response.

Materials & experimental systems

n/a	Involved in the study
☒	☐ Antibodies
☒	☐ Eukaryotic cell lines
☒	☐ Palaeontology and archaeology
☒	☐ Animals and other organisms
☒	☐ Clinical data
☒	☐ Dual use research of concern

Methods

n/a	Involved in the study
☒	☐ ChIP-seq
☒	☐ Flow cytometry
☒	☐ MRI-based neuroimaging
